# Reporting of patient-reported outcomes (PROs) in randomized controlled trials of anemia treatments for people with CKD: a scoping review

**DOI:** 10.1186/s41687-026-01007-2

**Published:** 2026-02-21

**Authors:** Dipal M. Patel, Renee F. Wilson, Troy Gharibani, Lisa M. Wilson, Xuhao Yang, Yuanxi Jia, Karen A. Robinson

**Affiliations:** 1 Division of Nephrology, Department of Medicine, Johns Hopkins University School of Medicine, 1830 E. Monument St, Suite 416, Baltimore, MD 21287 USA; 2 Department of Health Policy and Management, Johns Hopkins Bloomberg School of Public Health, Baltimore, MD USA; 3 Division of General Internal Medicine, Department of Medicine, Johns Hopkins University School of Medicine, Baltimore, MD USA; 4 Department of Epidemiology, Johns Hopkins Bloomberg School of Public Health, Baltimore, MD USA

**Keywords:** Anemia, Chronic kidney disease, Patient-reported outcomes (PROs), Patient-reported outcome measures (PROMs)

## Abstract

**Background:**

Patient-reported outcomes (PROs) are key outcomes of importance for people with chronic kidney disease (CKD), and may be of even higher relevance for patients with concomitant anemia which can often be symptomatic. Thus, PROs should be included as outcomes in clinical trials of interventions delivered to people with CKD. We evaluated the reporting of PROs in randomized controlled trials (RCTs) of anemia treatments delivered to people with CKD, assessing adherence to Consolidated Standards of Reporting Trials (CONSORT)-PRO reporting guidelines.

**Methodology:**

We conducted a scoping review in October 2024 using PubMed, Embase, and the Cochrane Central Register of Controlled Trials to identify RCTs of anemia treatments delivered to people with CKD, and subsequently identified publications of these RCTs which reported PROs data. We appraised adherence to seven elements outlined in CONSORT-PRO guidelines.

**Results:**

From 280 publications of 248 eligible RCTs, 41 publications (15%) reported PROs data for iron treatments (*n* = 16), erythropoiesis stimulating agents (ESAs; *n* = 22), hypoxia-inducible factor–prolyl hydroxylase inhibitors (HIF-PHIs; *n* = 2), or HIF-PHIs versus ESAs (*n* = 1). Data were reported for 22 different PRO measures. Most publications did not adhere to CONSORT-PRO guidelines, with 39% identifying the PRO measure in the study abstract, 7% providing a hypothesis surrounding the PROs, 10% reporting methods of PRO data collection, 27% providing statistical methods for handling missing data, and 10% discussing PRO-specific limitations and implications for generalizability. No publication followed all seven CONSORT-PRO elements evaluated.

**Conclusions:**

There are a limited number of publications reporting PROs from RCTs of anemia treatments delivered to people with CKD. PRO measures used, as well as methods of data reporting and analysis, are highly variable. These findings highlight the need to adhere to established guidelines for reporting PROs data, which will enable interpretability and reliability of data in caring for people with CKD and anemia.

**Supplementary Information:**

The online version contains supplementary material available at 10.1186/s41687-026-01007-2.

## Background

Patients with chronic kidney disease (CKD) have identified patient-reported outcomes (PROs), including fatigue, life participation, and quality of life, as being critically important in their experience of disease [1],. Assessing PROs is key to providing person-centered care, wherein each patient is empowered to report their experience of disease and receive a tailored management strategy specific to their needs [2, 3]. PRO assessment increases patient satisfaction [4, 5] and patient activation [6], and facilitates shared decision-making and self-management [7]. PROs can also be linked to epidemiologic measures [8] and can be used to triage patients at high risk for poor outcomes, as seen in oncology practices which use symptom-triggered, nurse-driven interventions to reduce hospitalization rates in people receiving cancer treatment [9–11].

Additionally, attention has been called to the need to include PROs as core outcomes in clinical nephrology trials [12, 13]. Inclusion of PROs as trial outcomes is especially vital when the intervention being delivered is presumed to impact aspects of health-related quality of life. As a prime example, patients with CKD often develop iron deficiency and anemia, the prevalence of which worsens as kidney disease progresses [14]. According to the U.S. Renal Data Systems 2024 annual report [15], ~20% of people with CKD G4 and ~37% of people with CKD G5 have anemia with a hemoglobin value < 10 g/dL. Not only is anemia associated with increased risk of CKD progression, cardiovascular events, and mortality, but it is also associated with fatigue and worsening physical function [16]. Therefore, treatment of anemia is often pursued as a mechanism to improve functionality and reduce symptoms. Several randomized clinical trials (RCTs) have been conducted to test the impact of different treatments for anemia, including iron, erythropoiesis stimulating agents (ESAs), and hypoxia-inducible factor prolyl hydroxylase inhibitors (HIF-PHIs), on laboratory markers of anemia as well as on clinical outcomes for people with CKD. The consistency and methods by which these treatments are evaluated for their impact on PROs is less clear.

The objective of this scoping review was to evaluate the reporting of PROs in RCTs of anemia treatments delivered to people with CKD. To evaluate the completeness of PRO measurement and data reporting in these trials, we assessed adherence to guidelines recommended by the Consolidated Standards of Reporting Trials (CONSORT)-PRO Extension [17].

## Methods

This scoping review builds on data obtained through systematic reviews conducted for Kidney Disease: Improving Global Outcomes (KDIGO) to support their updated guideline on management of anemia in CKD [16]. These systematic reviews synthesized data from RCTs of treatments for iron deficiency and anemia in people with CKD. Results are reported in accordance with Preferred Reporting Items for Systematic reviews and Meta-Analyses extension for Scoping Reviews (PRISMA-ScR) guidelines [18].

### Protocol and registration

Protocols for systematic reviews used for this research were registered on PROSPERO: CRD42023429159 (iron dosing), CRD42023429837 (ESAs), CRD42023429442 (HIF-PHIs), and CRD42023429837 (RCTs comparing HIF-PHIs with ESAs). A protocol for this scoping review was registered on the Open Science Framework (https://osf.io/gq7zu).

### Information sources and search strategy

In October 2024, we searched the following databases for publications of RCTs assessing anemia/iron deficiency treatments for people with CKD: PubMed, Embase, and the Cochrane Central Register of Controlled Trials (CENTRAL) (please see Supplemental Tables 1-9 for a list of keywords used). The search strategies were reviewed using the Peer Review of Electronic Search Strategies (PRESS) checklist [19]. We manually reviewed reference lists of included articles and relevant reviews.

**Table 1:** Key elements of the CONSORT-PRO extension [17] evaluated for each RCT included in analysis

Section	Recommended Content
Title and Abstract	Is the PRO measure identified in the abstract as a primary or secondary outcome?
Introduction	Is a background and rationale for measuring PROs included?
	Is there a hypothesis for relevant PRO domains?
Methods	Are methods of data collection (*e.g.*, paper, telephone, or electronic; self-administered versus provider-administered) provided?
Randomization	Are statistical methods for missing PROs data explicitly stated?
Results	Are the number of PROs data at baseline and subsequent time points transparent?
Discussion	Are PRO-specific limitations of study findings, as well as generalizability of results to other populations, discussed?

Role of the funding source

KDIGO supported the systematic reviews described here, including input on our initial research question and populations and outcomes of interest. KDIGO had no role in the scoping review presented in this manuscript

### Eligibility criteria

We included RCTs that compared the following anemia/iron deficiency treatment options for adults and children with CKD: (1) iron therapy (oral, IV, or dialysate) to other iron dosing modalities, placebo, or no iron therapy; (2) ESA therapy to other ESAs, other doses and routes of ESA, other hemoglobin thresholds/targets for ESA therapy, or placebo or no ESA therapy; (3) HIF-PHI therapy to other HIF-PHIs, other HIF-PHI doses, other hemoglobin thresholds/targets for HIF-PHI therapy, or placebo or no HIF-PHI therapy; (4) ESAs versus HIF-PHIs. CKD was defined as estimated glomerular filtration rate (eGFR) < 60 ml/min/1.73 m^2^ and/or albuminuria > 300 mg/g. We included data for people with CKD on dialysis and those who had received a kidney transplant. Study inclusion was not limited by language, timing, or setting. If a study had a mixed population, such as those with or without CKD, we included summary data from subgroups of those with CKD if available. We excluded grey literature.

Among publications of the RCTs eligible for the systematic reviews, we searched for publications which reported data gathered from validated PRO measures, and which evaluated the impact of anemia treatments on PROs. We excluded publications with clinician-reported measures which were not reported by patients (*e.g.*, quantitative measurements of muscle strength or exercise endurance assessed by a member of the clinical or research team).

### Selection process

Systematic reviews were conducted per methods reported elsewhere and included PROs as an outcome of interest [16]. For this scoping review, we identified the subset of publications from the systematic reviews which reported PROs data. We allowed multiple publications for each RCT, as long as the publication included PROs data.

### Data extraction and data items

We extracted key variables relevant to PRO measurement and analysis, including the PRO measure domains assessed in each publication, methods of collecting PRO data (*e.g.*, timepoints of assessment), methods of data analysis, and elements of data reporting (*e.g.*, number of measurements available at each timepoint). Data extraction was conducted by one reviewer and verified by a second reviewer.

### Data synthesis and appraisal

Given our evaluation of data reporting, we considered publications to be the unit of analysis. Data from each publication were considered independently. We appraised the adherence of each publication to seven relevant and quantifiable elements of the CONSORT-PRO extension (Table 1) [17]. Briefly, CONSORT (Consolidated Standards of Reporting Trials) guidelines provide recommendations for a minimum set of items to be included when reporting RCT data [20], and are endorsed by major medical journals. The CONSORT-PRO extension aims to improve completeness and transparency of reporting PROs in RCTs with attention devoted to specific methodologies of PRO data collection and analysis [17].

## Results

### Search results

**Fig. 1:**
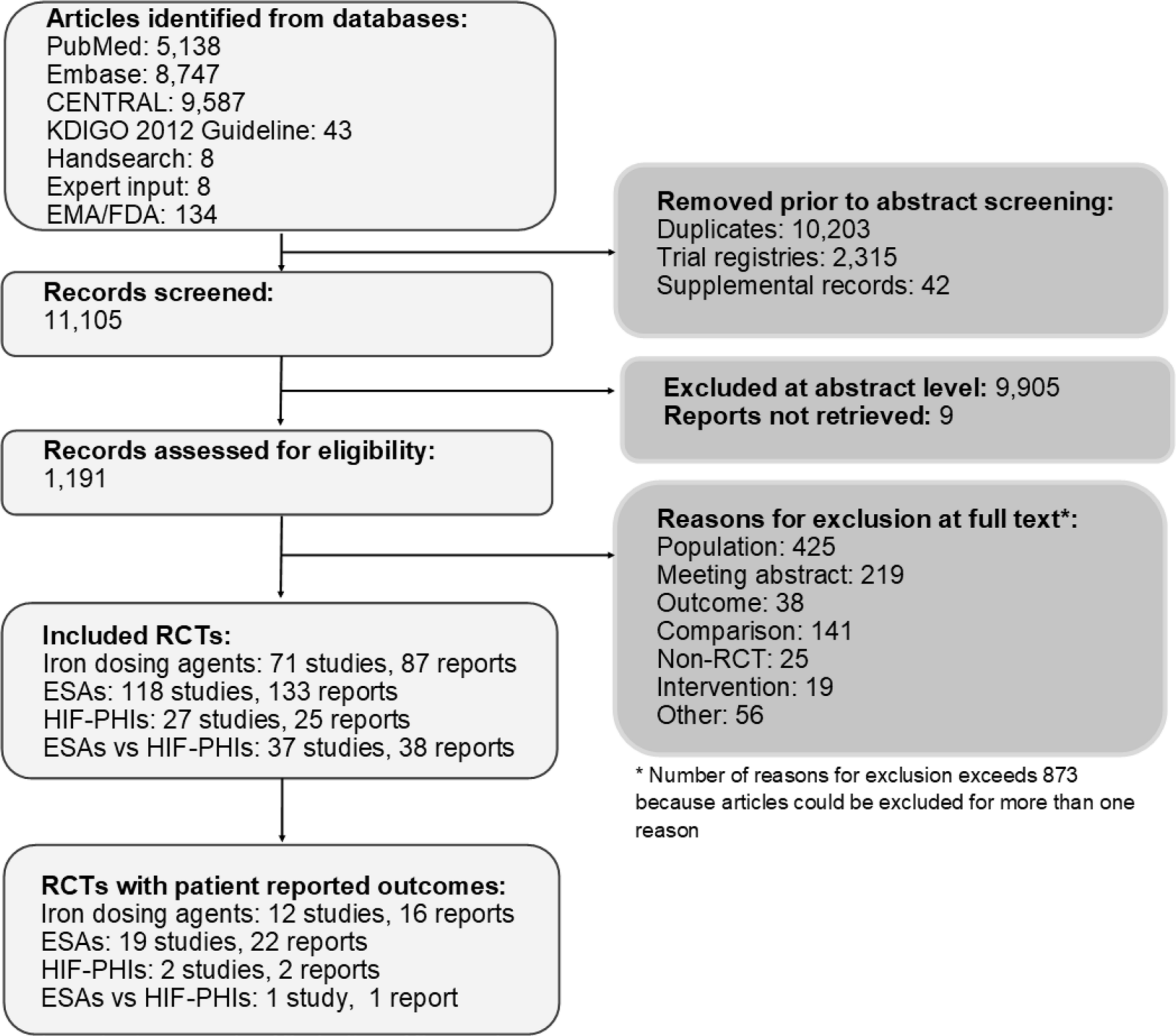
ESA: erythropoiesis stimulating agent; HIF-PHI: hypoxia-inducible factor prolyl hydroxylase inhibitors; PRO: patient-reported outcome; RCT: randomized controlled trial

We identified 248 eligible RCTs and 280 associated publications which reported effectiveness of iron dosing agents, ESAs, HIF-PHIs, or ESAs versus HIF-PHIs (Fig. 1, Supplemental Fig. 1-4).

We then identified publications which reported PROs data for people with CKD. In total, we identified 41 publications meeting eligibility criteria: 16 of 87 publications of RCTs studying iron therapy (18%), 22 of 133 publications of RCTs studying ESAs (17%), 2 of 25 publications of RCTs studying HIF-PHIs (8%), and 1 of 38 publications of RCTs comparing HIF-PHIs to ESAs (3%).

### Study characteristics

Characteristics of publications classified by treatment type are outlined in Tables 2-5. 16 publications reported PROs data from RCTs of iron therapies delivered to people with CKD (Table 2). Two of these publications reported PROs data from people on hemodialysis; otherwise, publications reported PROs data from people with non-dialysis-dependent CKD. Seven of the 16 publications reported data from RCTs of people with heart failure receiving iron therapies, with subgroup analyses of people with CKD.

**Table 2:** PROs reported in publications of RCTs studying iron therapies given to people with CKD

Publications of iron therapies delivered to people with CKD							
Publication	Population	Comparison	PRO measure(s) collected	Domain(s) reported in manuscript	Pre-specified timeline of assessments	Method of PROs data analysis	Conclusions reported in manuscript
Van Wyck et al., 2005 [21]	ND	IV iron (*n* = 79) v oral iron (*n* = 82)	SF-36	PF, RP, BP, GH, VT, SF, RE, MH	Not specified	Change from baseline to day 56	No significant difference between treatment groups
Agarwal et al., 2006 [22]	ND	IV iron (*n* = 36) v oral iron (*n* = 39)	KDQoL*	PHC, MHC, BKD, S/P, EKD	Baseline; day 43 or at early termination	Within-group change from baseline; Between-group differences	Significant improvement in within-group PHC scores for patients receiving IV iron; Significant improvement in S/P and EKD scores for patients receiving IV iron, compared to those receiving oral iron
Toblli et al., 2007 [23] and Toblli *et al.,* 2017 [24]	Heart failure (subgroup of patients with ND)	IV iron (*n* = 20) v saline (*n* = 20)	MLHFQ	n/a	Not specified	Within-group and between-group differences	Between-group significant difference favoring IV iron, at 6 months
			NYHA class				Within-group significant improvement in NYHA class for patients receiving IV iron, comparing baseline to 6 months; Between-group significant difference favoring IV iron, at 6 months and throughout 5-year follow-up
FAIR-HF: Anker et al., 2009 [25, 26], Comin-Colet et al., 2013 [27], and Ponikowski et al., 2015 [28]	Heart failure (subgroup of patients with ND)	IV iron (*n* = 130) v placebo (*n* = 73)	NYHA class (in [25, 26])	n/a	Baseline; weeks 4, 12, 24	Between-group differences using polytomous regression (NYHA model adjusted for baseline value)	Significant improvement for patients receiving IV iron
			PGA (in [25, 26])				
			KCCQ (in [27])	Overall summary score			
			EQ-5D VAS (in [28])				
FIND-CKD: Macdougall et al., 2014 [29, 30]	ND	IV iron (*n* = 305) v oral iron (*n* = 308)	SF-36	Not specified	Not specified	Not specified	No significant difference between treatment groups
Agarwal et al., 2015 [31]	ND	IV iron (*n* = 67) v oral iron (*n* = 69)	KDQoL*	PHC, MHC, E/F, CF	Randomization; 1, 2, 3, 6, 12, 18, 24 months	LSM change from baseline at specified timepoints	No significant difference between or within treatment groups
Kalra et al., 2016 [32]	ND	IV iron (*n* = 233) v oral iron (*n* = 118)	LASA	Energy level, daily activity, overall QoL	Baseline; 4, 8 weeks	LSM within-group and between-group change from baseline at 4 and 8 weeks	Significant within-group improvement in all domains from baseline to 8 weeks; No significant difference between treatment groups
Deng et al*.,* 2017 [33]	HD	IV iron (*n* = 16) v placebo (*n* = 16)	IRLS	Overall score	Baseline, 2 weeks	Change from baseline to 2 weeks	Decrease in IRLS was greater for patients in IV iron group than for patients in placebo group
PIVOTAL: Macdougall et al*.,* 2019 [34]	HD	High-dose IV iron (*n* = 1093) v low-dose IV iron (*n* = 1048)	EQ-5D*	QoL health index	Not specified	LSM change averaged over time	No significant difference between treatment groups
			KDQoL*	Overall score			
Bhandari et al., 2021 [35, 36]	ND	IV iron (*n* = 26) v placebo (*n* = 28)	NYHA class	n/a	Baseline; 1,3 months	Change from baseline at 1 and 3 months	No significant difference between treatment groups
			KDQoL*	Physical health, mental health, total score, VT			Modest improvement in scores for patients receiving IV iron (*p* > 0.05)
			MLHFQ	Not specified			No significant difference between treatment groups
AFFIRM-HF: Jankowska et al., 2021 [37] and Macdougall et al., 2023 [38]	Heart failure (subgroup of patients with CKD G3-5)	IV iron (*n* = 535) v placebo (*n* = 523)	KCCQ-12	OSS, CSS	Baseline; weeks 2, 4, 6, 12, 24, 36, 52	Within-group and between-group adjusted mean change from baseline	Non-significant improvement in OSS and CSS at 24 weeks, for patients receiving IV iron [37]; Significant between-group benefit in OSS and CSS at 4, 6, 12, and 24 weeks, for patients receiving IV iron compared to those receiving placebo [38]
Greenwood et al., 2023 [39, 40]	ND	IV iron (*n* = 37) v placebo (*n* = 38)	Chalder Fatigue	N/A	Baseline; weeks 4, 12	Change from baseline at 4 or 12 weeks	No significant difference between treatment groups
			KDQoL-SF 1.3	PHC, MHC, BKD, S/P, EKD			
			WSAS	N/A			

Populations are specified as: HD (hemodialysis) and ND (non-dialysis). *Additional details of PRO measures (*e.g.*, version of instrument used) were not specified. AFFIRM-AHF: A Randomised, Double-blind Placebo Controlled Trial Comparing the Effect of Intravenous Ferric Carboxymaltose on Hospitalisations and Mortality in Iron Deficient Subjects Admitted for Acute Heart Failure; LSM: least squares means; FAIR-HF: Ferric Carboxymaltose in Patients with Heart Failure and Iron Deficiency; FIND-CKD: Ferinject® assessment in patients with Iron deficiency anaemia and Non-Dialysis-dependent Chronic Kidney Disease; PIVOTAL: Proactive IV Iron Therapy in Haemodialysis Patients; QoL: quality of life

EQ-5D: European Quality of Life 5-Dimension

EQ-5D-5 L VAS: 5-Level visual analogue scale

IRLS: International Restless Legs Syndrome Study group Rating Scale for Restless Legs Syndrome

KCCQ: Kansas City Cardiomyopathy Questionnaire; domains include overall summary score (OSS) and clinical summary score (CSS)

KDQ: Kidney Disease Questionnaire; domains include physical symptoms (PS), fatigue (FA), depression (D), frustration (FR), and relationships with others (R)

KDQoL: Kidney Disease Quality of Life; domains include physical health composite (PHC), mental health composite (MHC), burden of kidney disease (BKD), symptoms/problems (S/P), effects of kidney disease (EKD), energy/fatigue (E/F), quality of social interaction (QSI), cognitive function (CF), sexual function (SX), sleep (SL)

KDQOL-SF 1.3: Short-Form version 1.3; domains include KDQoL and SF-36 domains listed below

LASA: Linear Analogue Self-Assessment

MLHFQ: Minnesota Living With Heart Failure® Questionnaire

NYHA Class: New York Heart Association Classification

PGA: Patient Global Assessment

SF-36: 36-Item Short-Form Survey; domains include physical functioning (PF), role-physical (RP), bodily pain (BP), general health (GH), vitality (VT), social functioning (SF), role-emotional (RE), mental health (MH); composite physical health score and mental health score

WSAS: Work and Social Adjustment Scale

22 publications reported PRO data from RCTs of ESA therapies delivered to people with CKD (Table 3). Most publications reported outcomes for adults with dialysis-dependent or non-dialysis-dependent CKD, with two publications reporting data from kidney transplant recipients, and one publication reporting data from children with CKD.

**Table 3:** PROs reported in publications of RCTs studying ESA therapies given to people with CKD

Publications of ESA therapies delivered to people with CKD							
Publication	Population	Comparison	PRO measure(s) collected	Domain(s) reported in manuscript	Pre-specified timeline of assessments	Method of PROs data analysis	Conclusions reported in manuscript
CanEPO: CanEPO study group [41] and Laupacis et al., 1990 [42]	HD	ESA with high Hb target (*n* = 38) v ESA with low Hb target (*n* = 40) v placebo (*n* = 40)	KDQ	PS, FA, D, FR, R	Baseline; months 2, 4, 6	Within-group and between-group differences from baseline	Greater improvement in PS, FA, D, and R scores for patients treated with ESA compared to patients treated with placebo; No significant difference between patients in high Hb v low Hb target group
			SIP	Overall, physical, psychological scores			Greater improvement in global and physical scores for patients treated with ESA compared to placebo
			TTO	Overall QOL			No significant difference between groups
Foley et al., 2000 [43]	HD	High Hb (*n* = 70) v low Hb (*n* = 72) target	KDQ	PS, FA, D, FR, R	Baseline; week 12, 24, 48 (week 12 KDQ was optional)	Repeated measures analysis of variance, comparing scores from baseline to those at 24 and 48 weeks	Significantly greater improvements in fatigue, depression, and relationships with others for patients in high Hb target group
			SF-36	PF, RP, BP, GH, VT, SF, RE, MH	Randomization and at 12, 24, and 48 weeks (planned); 12 week data were collected at the investigators discretion	Change from baseline at 24 and 48 weeks	No significant difference in scores over time, within-group or between-group
			HUI	Overall index of health	Randomization and at 12, 24, and 48 weeks	Not specified	
Furuland et al., 2003 [44]	HD	High Hb (*n* = 129) v low Hb (*n* = 124) target	KDQ	PS, FA, D, FR, R	Baseline, 1 year	Within-group and between-group difference from baseline at 48 weeks	Patients in high Hb target group had more improvement in symptoms; Significantly better between-group difference in scores for patients in high Hb target group
Roger et al., 2004 [45]	ND	High Hb (*n* = 75) v low Hb (*n* = 80) target	RQLP	Total score	Not specified	Difference from baseline at 2 years	No significant difference between groups
			SF-36	Physical health, mental health	Randomization and at 2 years		
Parfrey et al., 2005 [46] and Foley et al., 2009 [47]	HD	High Hb (*n* = 296) v low Hb (*n* = 330) target	KDQOL (containing SF-36)**	Reported in Parfrey et al. [46]): VT, QSI Reported in Foley et al. [47]: BKD, QSI, CF, S/P, EKD, SX, SL, PF, RP, BP, GH, RE, SF	Baseline; weeks 24, 36, 48, 60, 72, 84, 96	In Parfrey et al. [46]: Mean follow-up minus baseline score, with *p* values adjusted for multiple comparisons using the Hochberg procedure In Foley et al. [47]: Mixed modeling with estimated time-integrated QOL effects; Change from baseline between groups	In Parfrey et al. [46]: VT significantly higher for patients in high Hb group at weeks 24, 36, 48, 60, 72; No significant difference between groups for QSI or FACIT scores In Foley et al. [47]: E/F significantly higher for patients in high Hb group; No significant difference in other scores
			FACIT-fatigue (reported in Parfrey et al. [46])	Fatigue			
Rossert et al., 2006 [48]	ND	High Hb (*n* = 195) v low Hb (*n* = 195) target	SF-36	PF, RP, GH, VT, SF, MH	Baseline; every 9 months and at study termination	Between-group comparisons of scores using mixed-effect model	At end of stabilization phase: Significantly higher VT scores for patients in high Hb target group; Non-significant higher PF and RP scores for patients in high Hb target group; During maintenance phase: No significant between-group differences
CREATE: Drueke et al., 2006 [49]	ND	High Hb (*n* = 301) v low Hb (*n* = 302) target	SF-36	PF, RP, GH, VT, SF, MH	Not specified	Change from baseline at year 1 and year 2	At year 1, patients in normal Hb group had significantly higher improvement in scores in all domains; At year 2, patients in normal Hb group had significantly higher improvement in GH and VT scores
			NYHA class	n/a		Time to worsening of NYHA class	No significant different between groups
CHOIR: Singh et al., 2006 [50]	ND	High Hb (*n* = 715) v low Hb (*n* = 717) target	SF-36	PF, RP, GH, VT, SF, MH	Not specified	Change from baseline; ANCOVA with baseline score as independent cofactor	Significantly greater improvement in SF-36 RE for patients in low Hb group; Otherwise no significant difference between groups
			KDQ	Total score			
			LASA	Energy, activity, overall QoL			
Ritz et al., 2007 [51]	ND	High Hb (*n* = 88) v low Hb (*n* = 82) target	SF-36	PF, RP, BP, GH, VT, SF, RE, MH	Baseline, study end (15 months)	Mean total score at end of study, using ANCOVA with baseline score as independent cofactor	GH significantly better for patients in high Hb target; Data for other domains not presented
TREAT: Pfeffer et al., 2009 [52] and Lewis et al., 2011 [53]	ND	ESA (*n* = 2012) v placebo (*n* = 2026)	SF-36	PF, RP, BP, GH, VT, SF, RE, MH	Baseline; weeks 13, 25, 49, 73, 97, 121, 145, 169, 193, 217	Change from baseline at specified timepoints; Proportion achieving clinically meaningful change	Reported in Pfeffer et al., 2009 [52]: No significant difference between groups Reported in Lewis et al., 2011 [53]: At 25 weeks, no significant difference in scores between groups; ESA group had a significantly higher proportion of patients achieving clinically meaningful change in VT domain at 25 and 97 weeks, and in PF domain at 97 weeks
			EQ-5D VAS	n/a		Change from baseline at specified timepoints	Reported in Pfeffer et al., 2009 [52]: No significant difference between groups Reported in Lewis et al., 2011 [53]: At 25 and 97 weeks, greater degree of improvement for patients receiving ESA
			FACT-Fatigue	n/a	Randomization and at 13, 25, 49, 73, and 97 weeks	Change from baseline at specified timepoints; Proportion achieving clinically meaningful change	Reported in Pfeffer et al., 2009 [52]: Greater degree of improvement for patients receiving ESA Reported in Lewis et al., 2011 [53]: At 25 and 97 weeks, greater degree of improvement for patients receiving ESA; ESA group had a significantly higher proportion of patients achieving clinically meaningful change at 13, 25, 49, and 97 weeks
Choukroun et al., 2010 [54]	KT	High Hb (*n* = 63) v low Hb (*n* = 62) target	KTQ-25	F, U/F, A, E	Baseline and month 6	Adjusted means change from baseline	Significant improvement in within-group change from baseline for F scores, for patients with higher Hb target; otherwise, no significant differences
			SF-36	PF, RP, BP, GH, VT, SF, RE, MH	Randomization and at 6, 12 months	Adjusted means change from baseline at 12 months	Significant improvement in within-group change from baseline for GH, VT, PF, PR, MH, and SF scores, for patients with higher Hb target; otherwise, no significant differences
Akizawa et al., 2011 [55]	ND	High Hb (*n* = 161) v low Hb (*n* = 160) target	SF-36	PF, RP, BP, GH, VT, SF, RE, MH	Baseline and week 12	Between-group comparisons of change from baseline	Greater improvement in scores for patients in high Hb group (*p* < 0.05 for VT scores)
			FACIT	Fatigue			Both groups demonstrated improvement in scores, without significant difference between groups
Roger et al., 2014 [56]	ND	ESA (*n* = 28) v placebo (*n* = 23)	SF-36	PF, RP, BP, GH, VT, SF, RE, MH	Screening, baseline, weeks 12, 24, 36	Change from baseline at 24 weeks; Differences between groups after adjustment for baseline scores	Non-significantly improvement in VT scores for patients receiving ESA; Significant improvement in PF, RP, BP, GH, SF, RE, and MH scores for patients receiving ESA; No significant differences in VT scores between groups
			EQ-5D	Utility score	Randomization and at week 24	Change from baseline at 24 weeks	No significant difference between groups
			Fact-An	P, S, E, F, A, and total score	Randomization and at week 24	Change from baseline at 24 weeks	Significant improvement in F, A, and total score for patients receiving ESA
Oh et al., 2014 [57]	HD	ESA (*n* = 39) v ESA (*n* = 41)	SF-36 (Korean version)	PF, RP, BP, GH, VT, SF, RE, MH	Randomization; weeks 13, 25	Change from baseline	At week 13: decrease (worsening) of PF for patients receiving one ESA formulation; At week 25: improvement in all subscales except RE for patients receiving CERA
Satirapoj et al., 2014 [58]	HD	Liquid EPO (*n* = 19) v lyophilized EPO (*n* = 21)	SF-36	Physical health score, mental health score	Not specified	Change from baseline	No significant difference between or within treatment groups
Saglimbene et al., 2017 [59]	HD	High dose (*n* = 332) v low dose (*n* = 324) ESA	KDQOL-SF 1.3	PF, BKD, QSI, CF, PS, EKD, SL, overall health, RP, BP, GH, RE, MH, E/F, PSC, MHC	Baseline; months 6, 12	Difference between groups over time using multilevel regression	Significantly higher PF, RE, and PS domain scores favoring patients receiving low dose ESA; No significant differences in other domains
Satirapoj et al., 2017 [60]	HD	Liquid EPO (*n* = 30) v lyophilized EPO (*n* = 33)	SF-36	Physical health score, mental health score, total score	Baseline; week 24	Between-group and within-group change from baseline	No significant difference between or within treatment groups
Warady et al., 2018 [61]	Pediatric ND, HD, PD	Weekly (*n* = 59) v every 2 weeks (*n* = 56)	PedsQL	Total score, PF, EF, SF, ScF, PS	Baseline; weeks 13, 25	Change from baseline (planned for descriptive analysis only)	Small changes from baseline; largest improvements in parent-reported scores were in SF (weekly group) or PF (every 2 weeks group); largest improvements in patient-reported scores were in PF (weekly group) or EF (every 2 weeks group)
Pile et al., 2020 [62]	KT	ESA (*n* = 28) v placebo (*n* = 27)	SF-36	PF, RP, BP, GH, VT, SF, RE, MH	Not specified	Change from baseline at 24 weeks; Proportion of patients achieving minimal clinically importance difference	Significantly greater improvement in VT scores for patients treated with ESA; Significantly more patients achieving minimal clinically importance difference in MH domain for patients treated with ESA; No significant difference betewen groups for other domains

Populations are specified as: HD (hemodialysis), KT (kidney transplant), ND (non-dialysis), and PD (peritoneal dialysis). **Authors reported KDQOL and SF-36 domains and did not specify which version of KDQOL was used. CHOIR: Correction of Hemogloblin and Outcomes in Renal Insufficiency; CREATE: Cardiovascular Risk Reduction by Early Anemia Treatment with Epoetin Beta; ESA: erythropoiesis-stimulating agent; Hb: hemoglobin; LSM: least squares means; TREAT: Trial to Reduce Cardiovascular Events with Aranesp Therapy

EQ-5D: European Quality of Life 5-Dimension

EQ-5D-5 L VAS: 5-Level visual analogue scale

FACT-An: Functional Assessment of Cancer Therapy-Anemia; domains include physical (P), social (S), emotional (E), function (F), anemia (A), and total score

HRQoL: Health-Related Quality of Life

IRLS: International Restless Legs Syndrome Study group Rating Scale for Restless Legs Syndrome

KCCQ: Kansas City Cardiomyopathy Questionnaire; domains include overall summary score (OSS) and clinical summary score (CSS)

KDQ: Kidney Disease Questionnaire; domains include physical symptoms (PS), fatigue (FA), depression (D), frustration (FR), and relationships with others (R)

KDQoL: Kidney Disease Quality of Life; domains include physical health composite (PHC), mental health composite (MHC), burden of kidney disease (BKD), symptoms/problems (S/P), effects of kidney disease (EKD), energy/fatigue (E/F), quality of social interaction (QSI), cognitive function (CF), sexual function (SX), sleep (SL)

KDQOL-SF 1.3: Short-Form version 1.3; domains include KDQoL and SF-36 domains listed below

KTQ-25: Kidney Transplant Questionnaire-25; domains include physical symptoms (PS), fatigue (F), uncertainty/fear (U/F), appearance (A), and emotion (E)

MLHFQ: Minnesota Living With Heart Failure® Questionnaire

PedsQL: Pediatric Quality of Life Inventory; domains include physical functioning (PF), emotional functioning (EF), social functioning (SF), school functioning (ScF), and psychosocial composite score (PS)

PGIC: Patients’ Global Impression of Change

RLSS: Restless Leg Syndrome Scale

SF-36: 36-Item Short-Form Survey; domains include physical functioning (PF), role-physical (RP), bodily pain (BP), general health (GH), vitality (VT), social functioning (SF), role-emotional (RE), mental health (MH); composite physical health score and mental health score

SIP: Sickness Impact Profile; domains include physical score, psychosocial score, and overall score

TTO: Time Trade Off

WSAS: Work and Social Adjustment Scale

Two publications reported PROs data from people with non-dialysis-dependent CKD receiving HIF-PHI (Table 4), and one publication reported PROs data from people on dialysis who were randomized to receive either HIF-PHI or ESA (Table 5).

**Table 4:** PROs reported in publications of RCTs studying HIF-PHI therapies given to people with CKD

Publications of HIF-PHI therapies delivered to people with CKD							
Publication	Population	Comparison	PRO measure(s) collected	Domain(s) reported in manuscript	Pre-specified timeline of assessments	Method of PROs data analysis	Conclusions reported in manuscript
Coyne et al., 2021 [63]	ND	HIF-PHI (*n* = 616) v placebo (*n* = 306)	SF-36	PF, VT	Not specified	Difference from baseline to average score from weeks 12–28	Superiority of PF and VT scores for patients receiving HIF-PHI
Fishbane et al., 2021 [64]	ND	HIF-PHI (*n* = 1393) v placebo (*n* = 1388)	SF-36	PF, VT	Randomization; weeks 12, 28, 52	Adjusted LSM change from baseline to average score from weeks 12–28	Greater difference in PF and VT subscores for patients receiving HIF-PHI (*p* > 0.05)

HIF-PHI: hypoxia-inducible factor prolyl hydroxylase inhibitors; ND: non-dialysis. LSM: least squares means

SF-36: 36-Item Short-Form Survey; domains include physical functioning (PF), role-physical (RP), bodily pain (BP), general health (GH), vitality (VT), social functioning (SF), role-emotional (RE), mental health (MH); composite physical health score and mental health score

**Table 5:** PROs reported in publications of RCTs comparing HIF-PHI to ESA therapies given to people with CKD

Publications comparing HIF-PHI to ESA therapies delivered to people with CKD							
Publication	Population	Comparison	PRO measure(s) collected	Do(s) reported in manuscript	Pre-specified timeline of assessments	Method of PROs data analysis	Conclusions reported in manuscript
Csiky et al., 2021 [65]	HD or PD	HIF-PHI (*n* = 415) v ESA (*n* = 421)	SF-36	PF, VT	Baseline; weeks 8, 12, 28, 36, 52, and 76	Change in total score from baseline to average score from weeks 12–28	Roxadustat was superior to ESA for change in SF-36 PF and VT sub-scores
			EQ-5D-5 L VAS	Total score			No significant difference between treatment groups
			PGIC	Total score, HRQoL			No significant difference in total scores between treatment groups; more patients in HIF-PHI group reported improvement in HRQoL compared to patients in ESA group
			FACT-An	Total score			No significant difference between treatment groups

Populations are specified as: HD (hemodialysis) and PD (peritoneal dialysis)

EQ-5D: European Quality of Life 5-Dimension

EQ-5D-5 L VAS: 5-Level visual analogue scale

FACT-An: Functional Assessment of Cancer Therapy-Anemia; domains include physical (P), social (S), emotional (E), function (F), anemia (A), and total score

HRQoL: Health-Related Quality of Life

PGIC: Patients’ Global Impression of Change

SF-36: 36-Item Short-Form Survey; domains include physical functioning (PF), role-physical (RP), bodily pain (BP), general health (GH), vitality (VT), social functioning (SF), role-emotional (RE), mental health (MH); composite physical health score and mental health score

### PRO measures

**Fig. 2:**
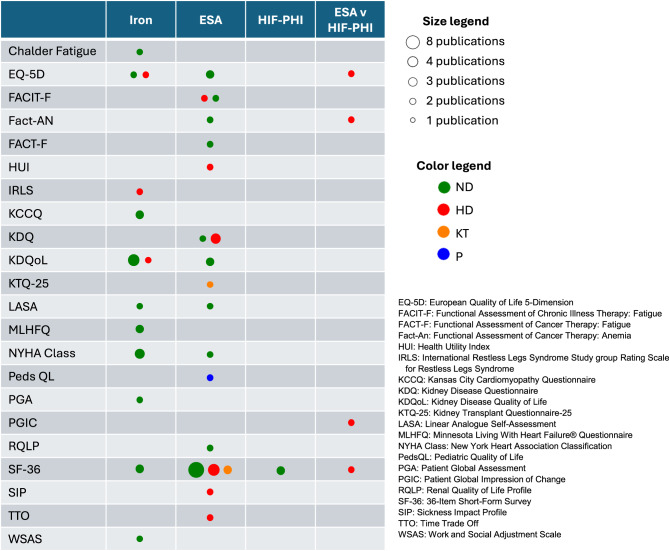
Evidence map displaying PRO measures reported in publications of anemia treatments delivered to people with CKD. DD: dialysis-dependent; ESA: erythropoiesis stimulating agent; HIF-PHI: hypoxia-inducible factor prolyl hydroxylase inhibitors; KT: kidney transplant recipient; ND: non-dialysis-dependent; P: pediatric patients; PRO: patient-reported outcome; RCT: randomized controlled trial

Among the 41 publications identified, 22 different PRO measures were used, with the most common being the Kidney Disease Quality-of-Life (KDQoL) and Short-Form 36 (SF-36) (Fig. 2). Most measures were global assessments, though publications may have reported limited data from these measures (Tables 2-5). For instance, 14 of the 22 publications studying ESAs reported data obtained from the SF-36, but some of these publications presented data from only specific domains of the SF-36, or stated only that there were no significant differences identified between treatment groups without providing detailed data from the measure.

In some cases, the same domain (*e.g.*, physical function) was evaluated using different PRO measures (Tables 2-5). Even when using the same PRO measure, publications often reported different methods of data analysis, including time points evaluated and comparator groups (*e.g.*, change from baseline, versus comparison between treatment groups).

### Adherence to CONSORT-PRO guidelines

**Fig. 3:**
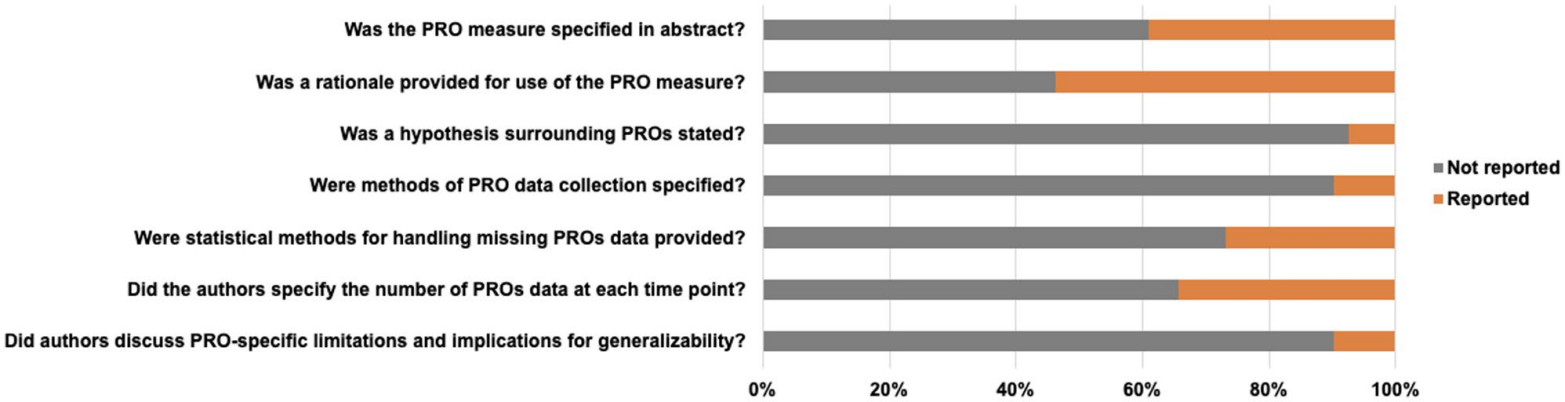
Adherence of publications reporting PROs data to key domains of CONSORT-PRO guidelines

Most publications did not adhere to CONSORT-PRO guidelines (Fig. 3, Supplemental Table 10). 39% of publications identified the PRO measure in the study abstract and 7% provided a hypothesis surrounding the PROs. Among the 41 publications, 10% reported methods of PRO data collection, 27% provided statistical methods for handling missing PROs data, and 34% specified the number of PROs data available at each time point. 10% of the publications discussed PRO-specific limitations and implications for generalizability. No publication followed all seven CONSORT-PRO guidelines, with a median of one element reported (range 0–6).

## Discussion

In this scoping review, we assessed adherence to CONSORT-PRO guidelines for publications of RCTs studying anemia treatments delivered to people with CKD. We identified 41 eligible publications (15% of eligible publications) which reported data from 22 different PRO measures. No publication reported all seven outlined elements of CONSORT-PRO guidelines, with the majority of publications missing key information including relevant hypotheses, methods of data collection and analysis, and implications for generalizability.

Based on our findings, there are several potential steps forward towards optimization of PROs data reporting for trials including people with CKD receiving anemia treatments. First, researchers in nephrology and other fields of medicine can continue to align studies with reporting guidelines, which list a minimum number of items that should be included to provide a transparent account of how the research was conducted and what was found [66]. Reporting guidelines for different study types, including updated CONSORT guidelines for randomized controlled trials [67], are collected and available from the Enhancing the QUAlity and Transparency Of health Research (EQUATOR) Network [68]. Use of CONSORT guidelines for nephrology RCTs has been recommended for over 15 years [69]. Unfortunately, a 2012 study by Fishbane et al. found that the majority of nephrology RCTs had insufficient reporting of randomization methodology [70]. Furthermore, a 2024 study by Crotty et al*. *identified that CONSORT guidelines were required for reporting of clinical trials by only 11 of 62 nephrology journals [71].

CONSORT-PRO guidelines were introduced in 2013 [17], but have similarly had suboptimal uptake. A systematic review of PROs in elderly patients with hip fracture found that 47.8% of studies examining PROs in this population satisfied < 50% of CONSORT-PRO criteria [72]. Among 88 RCTs with a validated PRO endpoint published in plastic surgery journals, adherence to CONSORT-PRO guidelines was < 40% [73]. A systematic review of PROs in trials of palliative radiotherapy found poor or moderate adherence to CONSORT-PRO guidelines, even when PROs were reported as a primary outcome [74]. A study of PROs data in cystic fibrosis noted that reporting completeness was higher for publications in journals requiring adherence to CONSORT guidelines [75], raising the possibility that PROs data reporting will improve as these guidelines are more widely adopted and potentially mandated.

Second, we draw attention to the need to establish standardized outcomes of interest, which can ensure that the appropriate PRO measures are consistently used and reported in publications of RCTs. The Core Outcome Measures in Effectiveness Trials (COMET) initiative brings together relevant stakeholders to develop sets of standardized “core outcome sets” relevant to specific conditions [76]. At the time of writing this manuscript, the COMET website lists one study which aims to develop core outcome sets for iron deficiency in anemia in pregnancy and postpartum stages. There are 24 studies which aim to develop core outcomes for people with dialysis-dependent or non-dialysis-dependent CKD, including studies led by the Standardized Outcomes in Nephrology (SONG) Initiative [77]. To our knowledge, there is no current study which aims to develop core outcomes specific to people with CKD and anemia. A core outcome set including PROs which is specific to people with CKD receiving treatment for anemia, developed in partnership with people with CKD, could then be incorporated into the design and execution of relevant RCTs.

These outcomes should also be accompanied by standard elements, as outlined in a study by Dickersin et al*.* [78]. After defining the domain of interest, the name of the instrument (*e.g.*, PRO measure) used to assess the domain, as well as proposed subscales to analyze, should be specified. Additional standard elements should include the unit of measurement for the outcome, the method of estimating the treatment effect (*e.g.*, individual-level change from baseline versus comparison of aggregate scores per treatment arm), and specified time points for analysis. As shown in our analyses, publications reporting PROs data for people with CKD receiving anemia treatments have not included these standard elements. Not only were different PRO measures used to assess the same outcome (*e.g.*, fatigue), but there was also a high degree of heterogeneity in the methods of analysis and time points reported. Similar heterogeneity was found in a recent systematic review of PRO measures for fatigue in patients with CKD [79].

In total, the heterogeneity in PRO evaluation as well as data reporting hinders the ability to understand the true impact of anemia treatments on PROs. Conclusions reported from publications of iron therapy and ESA treatments were variable, with some publications reporting that there was no significant difference in PROs between treatment groups, and others reporting improvement in specific domains of PRO measures. The two publications reporting data of HIF-PHI treatments reported improvement in domains of SF-36 for people receiving HIF-PHI. One publication which compared HIF-PHI to ESA treatment reported improvement in one of the four PRO measures used for people receiving HIF-PHI compared to ESA, with no significant difference in the other three PRO measures reported. Use of the same PRO measures and consistent methods of analysis would enable clinicians and patients to determine if anemia treatments could help address relevant symptoms.

Our study had some limitations. Analyses were limited to data available in publications, which in many cases may have been limited by reporting bias and/or selective outcome reporting (*e.g.*, studies which did not publish results from all PRO measure domains collected). Publications of anemia treatments which did not report subgroup analyses for people with CKD were not included. We did not evaluate adherence of publications to all elements outlined in the CONSORT-PRO extension [17], some of which were less quantifiable than elements outlined here.

Our study’s main strength is that it is among the few studies to evaluate the quality of PRO data reporting for people with CKD, who are known to face significant symptom burden. We appraised adherence of PROs data reporting to CONSORT-PRO guidelines which are increasingly recommended for use in reporting results of trials.

In conclusion, we identified a high degree of heterogeneity in reporting of PROs data for people with CKD receiving anemia treatments, not only in the PRO measure used but also in methods of data collection and analysis. Publications largely did not adhere to CONSORT-PRO guidelines. Incomplete data and variability in the reporting of PROs impacts our understanding of the true impact of anemia treatments on PROs reported by people with CKD. The ability of iron, ESA, and HIF-PHI treatments to improve aspects of health-related QoL warrant further investigation using standardized outcome measurements and reporting guidelines, only after which can the impact of these treatments on PROs truly be understood.

## Electronic supplementary material

Below is the link to the electronic supplementary material.

Supplementary Material 1

## Data Availability

Where not publicly available as part of the updated 2025 KDIGO Clinical Practice Guideline for Anemia in Chronic Kidney Disease, data supporting findings of this study will be made available upon request.
