# Supplementary material for: Reporting of patient-reported outcomes (PROs) in randomized controlled trials of anemia treatments for people with CKD: a scoping review
Source: J Patient Rep Outcomes. 2026 Feb 21;10:46. doi: 10.1186/s41687-026-01007-2 (PMC13031428; doi:10.1186/s41687-026-01007-2)
Supplement: Supplementary file 1 — Supplementary Material 1 [file 41687_2026_1007_MOESM1_ESM.docx]

**SUPPLEMENTAL FILES**

**Supplemental Table 1: PubMed search string for iron therapy review**

**Supplemental Table 2: Embase search string for iron therapy review**

**Supplemental Table 3: Cochrane search string for iron therapy review**

**Supplemental Table 4: PubMed search string for ESA review**

**Supplemental Table 5: Embase search string for ESA review**

**Supplemental Table 6: Cochrane Central search string for ESA review**

**Supplemental Table 7: PubMed search string for HIF-PHI review**

**Supplemental Table 8: Embase search string for HIF-PHI review**

**Supplemental Table 9: Cochrane Central search string for HIF-PHI review**

**Supplemental Figure 1. PRISMA diagram for identification of eligible publications for iron therapy review**

**Supplemental Figure 2. PRISMA diagram for identification of eligible publications for ESA review**

**Supplemental Figure 3. PRISMA diagram for identification of eligible publications for HIF-PHI review**

**Supplemental Figure 4. PRISMA diagram for identification of eligible publications for HIF-PHI versus ESA review**

**Supplemental Table 10: Reporting of CONSORT-PRO specifications in publications reporting PROs data**

**Supplemental Table 1. PubMed search string for iron therapy review**

| **#** | **Term** |
| --- | --- |
| 1 | “Iron dextran complex” [MeSH] |
| 2 | “Ferric compounds”[mh] |
| 3 | “Ferrous compounds”[mh] |
| 4 | “iron sulfate”[tiab] |
| 5 | “iron gluconate”[tiab] |
| 6 | “iron fumarate”[tiab] |
| 7 | “iron dextran”[tiab] |
| 8 | “iron sucrose”[tiab] |
| 9 | “iron saccharate”[tiab] |
| 10 | “ferric gluconate”[tiab] |
| 11 | “ferric compound”[tiab] |
| 12 | “ferric compounds”[tiab] |
| 13 | “ferric oxide”[tiab] |
| 14 | “ferrous gluconate”[tiab] |
| 15 | “ferrous bisglycinate”[tiab] |
| 16 | “ferumoxytol”[tiab] |
| 17 | “iron therapy”[tiab] |
| 18 | “iron therapies”[tiab] |
| 19 | “Iron supplement”[tiab] |
| 20 | “iron supplements”[tiab] |
| 21 | “iron supplementation”[tiab] |
| 22 | “iron supplementations”[tiab] |
| 23 | “IV iron”[tiab] |
| 24 | “intravenous iron”[tiab] |
| 25 | “iron infusion”[tiab] |
| 26 | “iron infusions”[tiab] |
| 27 | “Oral iron”[tiab] |
| 28 | “iron treatment”[tiab] |
| 29 | “iron treatments”[tiab] |
| 30 | iron[tiab] AND Dialysate[tiab] |
| 31 | #1 OR #2 OR #3 OR #4 OR #5 OR #6 OR #7 OR #8 OR #9 OR #10 OR #11 OR #12 OR #13 OR #14 OR #15 OR #16 OR #17 OR #18 OR #19 OR #20 OR #21 OR #22 OR #23 OR #24 OR #25 OR #26 OR #27 OR #28 OR #29 OR #30 |
| 32 | randomized controlled trial[pt] OR controlled clinical trial[pt] OR randomized[tiab] OR placebo[tiab] OR clinical trials as topic[mesh:noexp] OR randomly[tiab] OR trial[ti] |
| 33 | #31 AND #32 |
| 34 | (animals[mh] NOT humans [mh]) |
| 35 | #33 NOT #34 |

**Supplemental Table 2. Embase search string for iron therapy review**

| **#** | **Term** |
| --- | --- |
| 1 | “Iron dextran”/exp OR “iron dextran”:ti,ab |
| 2 | “Ferrous ion”/exp |
| 3 | “ferric ion”/exp |
| 4 | 'ferrous sulfate'/exp OR “iron sulfate”:ti,ab |
| 5 | 'ferrous gluconate'/exp OR “iron gluconate”:ti,ab OR “ferrous gluconate”:ti,ab |
| 6 | 'ferrous fumarate'/exp OR “iron fumarate”:ti,ab |
| 7 | “iron sucrose”:ti,ab |
| 8 | 'iron saccharate'/exp OR “iron saccharate”:ti,ab |
| 9 | 'ferric gluconate'/exp OR “ferric gluconate”:ti,ab |
| 10 | “ferric compound”:ti,ab OR “ferric compounds”:ti,ab |
| 11 | “ferric oxide”:ti,ab |
| 12 | “ferrous bisglycinate”:ti,ab |
| 13 | “ferumoxytol”:ti,ab |
| 14 | “Iron therapy”/exp OR “iron therapy”:ti,ab OR “iron therapies”:ti,ab |
| 15 | “Iron supplement”:ti,ab OR “iron supplements”:ti,ab OR “iron supplementation”:ti,ab OR “iron supplementations”:ti,ab |
| 16 | “IV iron”:ti,ab OR “intravenous iron”:ti,ab |
| 17 | “iron infusion”:ti,ab OR “iron infusions”:ti,ab |
| 18 | “Oral iron”:ti,ab |
| 19 | “iron treatment”:ti,ab OR “iron treatments”:ti,ab |
| 20 | Iron:ti,ab AND Dialysate:ti,ab |
| 21 | #1 OR #2 OR #3 OR #4 OR #5 OR #6 OR #7 OR #8 OR #9 OR #10 OR #11 OR #12 OR #13 OR #14 OR #15 OR #16 OR #17 OR #18 OR #19 OR #20 |
| 22 | random*:ab,ti OR placebo*:de,ab,ti OR (double NEXT/1 blind*):ab,ti |
| 23 | #21 AND #22 |
| 24 | (animals/exp NOT humans/exp) |
| 25 | #23 NOT #24 |
| 26 | ‘conference abstract’/it |
| 27 | #25 NOT #26 |

**Supplemental Table 3. Cochrane search string for iron therapy review**

| **#** | **Term** |
| --- | --- |
| 1 | MeSH descriptor: [Iron-Dextran Complex] explode all trees |
| 2 | MeSH descriptor: [Ferrous Compounds] explode all trees |
| 3 | MeSH descriptor: [Ferric Compounds] explode all trees |
| 4 | “iron sulfate”:ti,ab |
| 5 | 'ferrous sulfate':ti,ab |
| 6 | “iron gluconate”:ti,ab |
| 7 | 'ferrous gluconate’:ti,ab |
| 8 | “iron fumarate”:ti,ab |
| 9 | 'ferrous fumarate':ab,ti |
| 10 | “iron dextran”:ti,ab |
| 11 | “iron sucrose”:ti,ab |
| 12 | “iron saccharate”:ti,ab |
| 13 | “ferric gluconate”:ti,ab |
| 14 | “ferric compounds”:ti,ab |
| 15 | “ferric oxide”:ti,ab |
| 16 | “ferrous gluconate”:ti,ab |
| 17 | “ferrous bisglycinate”:ti,ab |
| 18 | “ferumoxytol”:ti,ab |
| 19 | “iron therapy”:ti,ab |
| 20 | “iron therapies”:ti,ab |
| 21 | “Iron supplement”:ti,ab |
| 22 | “iron supplements”:ti,ab |
| 23 | “iron supplementation”:ti,ab |
| 24 | “iron supplementations”:ti,ab |
| 25 | “IV iron”:ti,ab |
| 26 | “intravenous iron”:ti,ab |
| 27 | “iron infusion”:ti,ab |
| 28 | “iron infusions”:ti,ab |
| 29 | “Oral iron”:ti,ab |
| 30 | “iron treatment”:ti,ab |
| 31 | “iron treatments”:ti,ab |
| 32 | Iron:ti,ab AND Dialysate:ti,ab |
| 33 | #1 OR #2 OR #3 OR #4 OR #5 OR #6 OR #7 OR #8 OR #9 OR #10 OR #11 OR #12 OR #13 OR #14 OR #15 OR #16 OR #17 OR #18 OR #19 OR #20 OR #21 OR #22 OR #23 OR #24 OR #25 OR #26 OR #27 OR #28 OR #29 OR #30 OR #31 OR #32 in trials |

**Supplemental Table 4. PubMed search string for ESA review**

| **#** | **Term** |
| --- | --- |
| 1 | “Renal Insufficiency, Chronic”[mh] |
| 2 | “kidney glomerulus”[mh] |
| 3 | “Kidney Diseases”[mh] |
| 4 | “Chronic kidney disease”[tiab] |
| 5 | CKD[tiab] |
| 6 | “Chronic renal disease”[tiab] |
| 7 | "end-stage kidney disease"[tiab] |
| 8 | "end-stage renal disease"[tiab] |
| 9 | ESRD[tiab] |
| 10 | ESKD[tiab] |
| 11 | ESKF[tiab] |
| 12 | ESRF[tiab] |
| 13 | "chronic renal failure"[tiab] |
| 14 | "Renal replacement therapy"[Mesh] |
| 15 | “Sorption Detoxification”[mh] |
| 16 | “renal dialysis”[mh] |
| 17 | “kidney transplantation”[mh] |
| 18 | hemodialysis[tiab] |
| 19 | "peritoneal dialysis"[tiab] |
| 20 | ultrafiltration[mh] |
| 21 | “ultrafiltration”[tiab] |
| 22 | Hemofiltration[tiab] |
| 23 | Haemofiltration[tiab] |
| 24 | Hemodiafiltration[tiab] |
| 25 | Haemodiafiltration[tiab] |
| 26 | dialysis[tiab] |
| 27 | haemodialysis[tiab] |
| 28 | sorbtion[tiab] |
| 29 | detoxification[tiab] |
| 30 | (Transplant[tiab] OR transplants[tiab] OR graft[tiab] OR grafts[tiab] OR grafting[tiab]) AND (kidney[tiab] OR renal[tiab]) |
| 31 | #1 OR #2 OR #3 OR #4 OR #5 OR #6 OR #7 OR #8 OR #9 OR #10 OR #11 OR #12 OR #13 OR #14 #15 OR #16 OR #17 OR #18 OR #19 OR #20 OR #21 OR #22 OR #23 OR #24 OR #25 OR #26 OR #27 OR #28 OR #29 OR #30 |
| 32 | Erythropoietin[mh] |
| 33 | “recombinant erythropoietin”[tiab] |
| 34 | “Hematinics”[mh] |
| 35 | "continuous erythropoietin receptor activator" [Supplementary Concept] |
| 36 | “methoxy polyethylene glycol-epoetin beta”[tiab] |
| 37 | CERA[tiab] |
| 38 | “erythropoietin receptor activator”[tiab] |
| 39 | Epoetin[tiab] |
| 40 | epogen[tiab] |
| 41 | Erythropoietin[tiab] |
| 42 | “erythrocyte stimulat*”[tiab] |
| 43 | darbepoetin[tiab] |
| 44 | epokine[tiab] |
| 45 | procrit[tiab] |
| 46 | eprex[tiab] |
| 47 | Dynepro[tiab] |
| 48 | Epomax[tiab] |
| 49 | Hemax[tiab] |
| 50 | Silapo[tiab] |
| 51 | Retacrit[tiab] |
| 52 | Aranesp[tiab] |
| 53 | Epo[tiab] |
| 54 | Mircera[tiab] |
| 55 | ESA[tiab] |
| 56 | Erythropoiesis[tiab] |
| 57 | #32 OR #33 OR #34 OR #35 OR #36 OR #37 OR #38 OR #39 OR #40 OR #41 OR #42 OR #43 OR #44 OR #45 OR #46 OR #47 OR #48 OR #49 OR #50 OR #51 OR #52 OR #53 OR #54 OR #55 OR #56 |
| 58 | “Randomized controlled trial”[pt] OR “controlled clinical trial”[pt] OR randomized[tiab] OR placebo[tiab] “clinical trial”[pt] OR randomly[tiab] OR trial[tiab] OR “random allocation”[tiab] OR “double-blind method”[tiab] OR “single-blind method”[tiab] OR “clinical trial”[tiab] OR “clinical trial”[tiab] |
| 59 | #31 AND #57 AND #58 |
| 60 | Animals[mh] NOT humans[mh] |
| 61 | #59 NOT #60 |
| 62 | Date limited: October 2009 to present |

**Supplemental Table 5. Embase search string for ESA review**

| **#** | **Term** |
| --- | --- |
| 1 | “chronic kidney failure”/exp |
| 2 | “glomerulus”/exp |
| 3 | “Kidney Disease”/exp |
| 4 | “Chronic kidney disease”:ti,ab |
| 5 | CKD:ti,ab |
| 6 | “Chronic renal disease”:ti,ab |
| 7 | "end-stage kidney disease":ti,ab |
| 8 | "end-stage renal disease":ti,ab |
| 9 | ESRD:ti,ab |
| 10 | ESKD:ti,ab |
| 11 | ESKF:ti,ab |
| 12 | ESRF:ti,ab |
| 13 | "chronic renal failure":ti,ab |
| 14 | "Renal replacement therapy”/exp |
| 15 | “Sorption Detoxification”/exp |
| 16 | “hemodialysis”/exp |
| 17 | “kidney transplantation”/exp |
| 18 | Hemodialysis:ti,ab |
| 19 | "peritoneal dialysis":ti,ab |
| 20 | Ultrafiltration/exp |
| 21 | “ultrafiltration”:ti,ab |
| 22 | Hemofiltration:ti,ab |
| 23 | Haemofiltration:ti,ab |
| 24 | Hemodiafiltration:ti,ab |
| 25 | Haemodiafiltration:ti,ab |
| 26 | Dialysis:ti,ab |
| 27 | Haemodialysis:ti,ab |
| 28 | Sorbtion:ti,ab |
| 29 | Detoxification:ti,ab |
| 30 | (Transplant:ti,ab OR transplants:ti,ab OR graft:ti,ab OR grafts:ti,ab OR grafting:ti,ab) AND (kidney:ti,ab OR renal:ti,ab) |
| 31 | #1 OR #2 OR #3 OR #4 OR #5 OR #6 OR #7 OR #8 OR #9 OR #10 OR #11 OR #12 OR #13 OR #14 #15 OR #16 OR #17 OR #18 OR #19 OR #20 OR #21 OR #22 OR #23 OR #24 OR #25 OR #26 OR #27 OR #28 OR #29 OR #30 |
| 32 | Erythropoietin/exp |
| 33 | “recombinant erythropoietin”:ti,ab |
| 34 | “antianemic agent”/exp |
| 35 | "continuous erythropoietin receptor activator"/exp |
| 36 | “methoxy polyethylene glycol-epoetin beta”:ti,ab |
| 37 | CERA:ti,ab |
| 38 | “erythropoietin receptor activator”:ti,ab |
| 39 | Epoetin:ti,ab |
| 40 | Epogen:ti,ab |
| 41 | Erythropoietin:ti,ab |
| 42 | “erythrocyte stimulat*”:ti,ab |
| 43 | Darbepoetin:ti,ab |
| 44 | Epokine:ti,ab |
| 45 | Procrit:ti,ab |
| 46 | Eprex:ti,ab |
| 47 | Dynepro:ti,ab |
| 48 | Epomax:ti,ab |
| 49 | Hemax:ti,ab |
| 50 | Silapo:ti,ab |
| 51 | Retacrit:ti,ab |
| 52 | Aranesp:ti,ab |
| 53 | Epo:ti,ab |
| 54 | Mircera:ti,ab |
| 55 | ESA:ti,ab |
| 56 | Erythropoiesis:ti,ab |
| 57 | #32 OR #33 OR #34 OR #35 OR #36 OR #37 OR #38 OR #39 OR #40 OR #41 OR #42 OR #43 OR #44 OR #45 OR #46 OR #47 OR #48 OR #49 OR #50 OR #51 OR #52 OR #53 OR #54 OR #55 OR #56 |
| 58 | random*:ab,ti OR placebo*:de,ab,ti OR (double NEXT/1 blind*):ab,ti |
| 59 | #31 AND #57 AND #58 |
| 60 | Animals/exp NOT humans/exp |
| 61 | #59 NOT #60 |
| 62 | Date limited: 2009 to present |

**Supplemental Table 6. Cochrane Central search string for ESA review**

| **#** | **Term** |
| --- | --- |
| 1 | MeSH descriptor: [Renal Insufficiency, Chronic] explode all trees |
| 2 | MeSH descriptor: [Kidney Glomerulus] explode all trees |
| 3 | MeSH descriptor: [Kidney Diseases] explode all trees |
| 4 | “Chronic kidney disease”:ti,ab |
| 5 | CKD:ti,ab |
| 6 | “Chronic renal disease”:ti,ab |
| 7 | "end-stage kidney disease":ti,ab |
| 8 | "end-stage renal disease":ti,ab |
| 9 | ESRD:ti,ab |
| 10 | ESKD:ti,ab |
| 11 | ESKF:ti,ab |
| 12 | ESRF:ti,ab |
| 13 | "chronic renal failure":ti,ab |
| 14 | MeSH descriptor: [Renal Replacement Therapy] explode all trees |
| 15 | MeSH descriptor: [Sorption Detoxification] explode all trees |
| 16 | MeSH descriptor: [Renal Dialysis] explode all trees |
| 17 | MeSH descriptor: [Kidney Transplantation] explode all trees |
| 18 | Hemodialysis:ti,ab |
| 19 | "peritoneal dialysis":ti,ab |
| 20 | MeSH descriptor: [Ultrafiltration] 1 tree(s) exploded |
| 21 | “ultrafiltration”:ti,ab |
| 22 | Hemofiltration:ti,ab |
| 23 | Haemofiltration:ti,ab |
| 24 | Hemodiafiltration:ti,ab |
| 25 | Haemodiafiltration:ti,ab |
| 26 | Dialysis:ti,ab |
| 27 | Haemodialysis:ti,ab |
| 28 | Sorbtion:ti,ab |
| 29 | Detoxification:ti,ab |
| 30 | (Transplant:ti,ab OR transplants:ti,ab OR graft:ti,ab OR grafts:ti,ab OR grafting:ti,ab) AND (kidney:ti,ab OR renal:ti,ab) |
| 31 | #1 OR #2 OR #3 OR #4 OR #5 OR #6 OR #7 OR #8 OR #9 OR #10 OR #11 OR #12 OR #13 OR #14 #15 OR #16 OR #17 OR #18 OR #19 OR #20 OR #21 OR #22 OR #23 OR #24 OR #25 OR #26 OR #27 OR #28 OR #29 OR #30 |
| 32 | MeSH descriptor: [Erythropoietin] explode all trees |
| 33 | “recombinant erythropoietin”:ti,ab |
| 34 | MeSH descriptor: [Hematinics] explode all trees |
| 35 | "continuous erythropoietin receptor activator"/exp |
| 36 | “methoxy polyethylene glycol-epoetin beta”:ti,ab |
| 37 | CERA:ti,ab |
| 38 | “erythropoietin receptor activator”:ti,ab |
| 39 | Epoetin:ti,ab |
| 40 | Epogen:ti,ab |
| 41 | Erythropoietin:ti,ab |
| 42 | “erythrocyte stimulation”:ti,ab, word variations have been searced |
| 43 | Darbepoetin:ti,ab |
| 44 | Epokine:ti,ab |
| 45 | Procrit:ti,ab |
| 46 | Eprex:ti,ab |
| 47 | Dynepro:ti,ab |
| 48 | Epomax:ti,ab |
| 49 | Hemax:ti,ab |
| 50 | Silapo:ti,ab |
| 51 | Retacrit:ti,ab |
| 52 | Aranesp:ti,ab |
| 53 | Epo:ti,ab |
| 54 | Mircera:ti,ab |
| 55 | ESA:ti,ab |
| 56 | Erythropoiesis:ti,ab |
| 57 | #32 OR #33 OR #34 OR #35 OR #36 OR #37 OR #38 OR #39 OR #40 OR #41 OR #42 OR #43 OR #44 OR #45 OR #46 OR #47 OR #48 OR #49 OR #50 OR #51 OR #52 OR #53 OR #54 OR #55 OR #56 |
| 58 | #31 AND #57 |
| 59 | Date limited: Oct 2009 to present |

**Supplemental Table 7. PubMed search string for HIF-PHI review**

| **#** | **Term** |
| --- | --- |
| 1 | "Hypoxia-Inducible Factor-Proline Dioxygenases"[mh] |
| 2 | "Hypoxia-Inducible Factor-Proline Dioxygenases"[tiab] |
| 3 | "Prolyl-Hydroxylase Inhibitors"[mh] |
| 4 | "Prolyl-Hydroxylase Inhibitors”[tiab] |
| 5 | "Prolyl-Hydroxylase Inhibitor”[tiab] |
| 6 | "HIF prolyl-hydroxylase inhibitor"[tiab] |
| 7 | "HIF prolyl-hydroxylase inhibitors"[tiab] |
| 8 | “hypoxia-inducible factor stabilizer”[tiab] |
| 9 | “Hypoxia-inducible factor–prolyl hydroxylase inhibitors”[tiab] |
| 10 | "HIF-PHI"[tiab] OR “HIF-PHIs”[tiab] |
| 11 | “Hypoxia-inducible factors”[tiab] OR “Hypoxia-inducible factor”[tiab] |
| 12 | Daprodustat[tiab] |
| 13 | Desidustat[tiab] |
| 14 | Enarodustat[tiab] |
| 15 | Molidustat[tiab] |
| 16 | Vadadustat[tiab] |
| 17 | Roxadustat[tiab] |
| 18 | "GSK1278863" [Supplementary Concept] |
| 19 | "desidustat" [Supplementary Concept] |
| 20 | "enarodustat" [Supplementary Concept] |
| 21 | "molidustat" [Supplementary Concept] |
| 22 | "vadadustat" [Supplementary Concept] |
| 23 | “roxadustat” [Supplementary Concept] |
| 24 | #1 OR #2 OR #3 OR #4 OR #5 OR #6 OR #7 OR #8 OR #9 OR #10 OR #11 OR #12 OR #13 OR #14 OR #15 OR #16 OR #17 OR #18 OR #19 OR #20 OR #21 OR #22 OR #23 |
| 25 | “Renal Insufficiency, Chronic”[mh] |
| 26 | “kidney glomerulus”[mh] |
| 27 | “Kidney Diseases”[mh] |
| 28 | “Chronic kidney disease”[tiab] |
| 29 | CKD[tiab] |
| 30 | “Chronic renal disease”[tiab] |
| 31 | "end-stage kidney disease"[tiab] |
| 32 | "end-stage renal disease"[tiab] |
| 33 | ESRD[tiab] |
| 34 | ESKD[tiab] |
| 35 | ESKF[tiab] |
| 36 | ESRF[tiab] |
| 37 | "chronic renal failure"[tiab] |
| 38 | "Renal replacement therapy"[Mesh] |
| 39 | “Sorption Detoxification”[mh] |
| 40 | “renal dialysis”[mh] |
| 41 | “kidney transplantation”[mh] |
| 42 | hemodialysis[tiab] |
| 43 | "peritoneal dialysis"[tiab] |
| 44 | ultrafiltration[mh] |
| 45 | “ultrafiltration”[tiab] |
| 46 | Hemofiltration[tiab] |
| 47 | Haemofiltration[tiab] |
| 48 | Hemodiafiltration[tiab] |
| 49 | Haemodiafiltration[tiab] |
| 50 | dialysis[tiab] |
| 51 | haemodialysis[tiab] |
| 52 | sorbtion[tiab] |
| 53 | detoxification[tiab] |
| 54 | (Transplant[tiab] OR transplants[tiab] OR graft[tiab] OR grafts[tiab] OR grafting[tiab]) AND (kidney[tiab] OR renal[tiab]) |
| 55 | #25 OR #26 OR #27 OR #27 OR #28 OR #29 OR #30 OR #31 OR #32 OR #33 OR #34 OR #35 OR #36 OR #37 OR #38 OR #39 OR #40 OR #41 OR #42 OR #43 OR #44 OR #45 OR #46 OR #47 OR #48 OR #49 OR #50 OR #51 OR #52 OR #53 OR #54 |
| 56 | #24 AND #55 |
| 57 | randomized controlled trial[pt] OR controlled clinical trial[pt] OR randomized[tiab] OR placebo[tiab] OR clinical trials as topic[mesh:noexp] OR randomly[tiab] OR trial[ti] |
| 58 | #56 AND #57 |
| 59 | Animals[mh] NOT humans[mh] |
| 60 | #58 NOT #60 |

**Supplemental Table 8. Embase search string for HIF-PHI review**

| **#** | **Term** |
| --- | --- |
| 1 | "hypoxia inducible factor proline dioxygenase"/exp |
| 2 | "Prolyl-Hydroxylase Inhibitor"/exp |
| 3 | "Prolyl Hydroxylase Inhibitor":ti,ab |
| 4 | "Prolyl Hydroxylase Inhibitors":ti,ab |
| 5 | "HIF prolyl-hydroxylase inhibitor":ti,ab |
| 6 | "HIF prolyl-hydroxylase inhibitors":ti,ab |
| 7 | “hypoxia-inducible factor stabilizer”:ti,ab |
| 8 | “hypoxia-inducible factor stabilizers”:ti,ab |
| 9 | “Hypoxia-inducible factor–prolyl hydroxylase inhibitors”:ti,ab |
| 10 | “Hypoxia-inducible factor–prolyl hydroxylase inhibitor”:ti,ab |
| 11 | “Hypoxia inducible factor prolyl hydroxylase inhibitor”/exp |
| 12 | "HIF-PHI":ti,ab OR “HIF-PHIs”:ti,ab |
| 13 | “Hypoxia-inducible factors”:ti,ab OR “Hypoxia-inducible factor”:ti,ab |
| 14 | Daprodustat:ti,ab |
| 15 | Desidustat:ti,ab |
| 16 | Enarodustat:ti,ab |
| 17 | Molidustat:ti,ab |
| 18 | Vadadustat:ti,ab |
| 19 | Roxadustat:ti,ab |
| 20 | "Daprodustat”/exp |
| 21 | "desidustat"/exp |
| 22 | "enarodustat”/exp |
| 23 | "molidustat"/exp |
| 24 | "vadadustat"/exp |
| 25 | “roxadustat”/exp |
| 26 | #1 OR #2 OR #3 OR #4 OR #5 OR #6 OR #7 OR #8 OR #9 OR #10 OR #11 OR #12 OR #13 OR #14 OR #15 OR #16 OR #17 OR #18 OR #19 OR #20 OR #21 OR #22 OR #23 OR #24 OR #25 |
| 27 | “chronic kidney failure”/exp |
| 28 | “glomerulus”/exp |
| 29 | “Kidney Disease”/exp |
| 30 | “Chronic kidney disease”:ti,ab |
| 31 | CKD:ti,ab |
| 32 | “Chronic renal disease”:ti,ab |
| 33 | "end-stage kidney disease":ti,ab |
| 34 | "end-stage renal disease":ti,ab |
| 35 | ESRD:ti,ab |
| 36 | ESKD:ti,ab |
| 37 | ESKF:ti,ab |
| 38 | ESRF:ti,ab |
| 39 | "chronic renal failure":ti,ab |
| 40 | "Renal replacement therapy”/exp |
| 41 | “Sorption Detoxification”/exp |
| 42 | “hemodialysis”/exp |
| 43 | “kidney transplantation”/exp |
| 44 | Hemodialysis:ti,ab |
| 45 | "peritoneal dialysis":ti,ab |
| 46 | Ultrafiltration/exp |
| 47 | “ultrafiltration”:ti,ab |
| 48 | Hemofiltration:ti,ab |
| 49 | Haemofiltration:ti,ab |
| 50 | Hemodiafiltration:ti,ab |
| 51 | Haemodiafiltration:ti,ab |
| 52 | Dialysis:ti,ab |
| 53 | Haemodialysis:ti,ab |
| 54 | Sorbtion:ti,ab |
| 55 | Detoxification:ti,ab |
| 56 | (Transplant:ti,ab OR transplants:ti,ab OR graft:ti,ab OR grafts:ti,ab OR grafting:ti,ab) AND (kidney:ti,ab OR renal:ti,ab) |
| 57 | #27 OR #28 OR #29 OR #30 OR #31 OR #32 Or #33 OR #34 OR #35 OR #36 OR #37 OR #38 OR #39 OR #40 OR #41 OR #42 OR #43 OR #44 OR #45 OR #46 OR #47 OR #48 OR #49 OR #50 OR #51 OR #52 OR #53 OR #54 OR #55 OR #56 |
| 58 | #26 AND #57 |
| 59 | ‘Randomized controlled trial’/exp OR ‘controlled clinical trial’/exp OR randomized:ti,ab OR placebo:ti,ab ‘clinical trial’/exp OR randomly:ti,ab OR trial:ti,ab OR ‘random allocation’:ti,ab OR ‘double-blind method’:ti,ab OR ‘single-blind method’:ti,ab OR ‘clinical trial’:ti,ab |
| 60 | #26 AND |
| 61 | Animals/exp NOT humans/exp |
| 62 | #73 NOT #74 |

**Supplemental Table 9. Cochrane Central search string for HIF-PHI review**

| **#** | **Term** |
| --- | --- |
| 1 | MeSH descriptor: [Hypoxia-Inducible Factor-Proline Dioxygenases] explode all trees |
| 2 | MeSH descriptor: [Prolyl-Hydroxylase Inhibitors] explode all trees |
| 3 | "Prolyl Hydroxylase Inhibitor":ti,ab |
| 4 | "Prolyl Hydroxylase Inhibitors":ti,ab |
| 5 | "HIF prolyl-hydroxylase inhibitor":ti,ab |
| 6 | "HIF prolyl-hydroxylase inhibitors":ti,ab |
| 7 | “hypoxia inducible factor stabilizers”:ti,ab |
| 8 | "HIF-PHI":ti,ab OR “HIF-PHIs”:ti,ab |
| 9 | "HIF PHI":ti,ab OR “HIF PHIs”:ti,ab |
| 10 | “Hypoxia inducible factors”:ti,ab OR “Hypoxia inducible factor”:ti,ab |
| 11 | Daprodustat:ti,ab |
| 12 | Desidustat:ti,ab |
| 13 | Enarodustat:ti,ab |
| 14 | Molidustat:ti,ab |
| 15 | Vadadustat:ti,ab |
| 16 | Roxadustat:ti,ab |
| 17 | (FG-4592):ti,ab |
| 18 | ("BAY-85 3934"):ti,ab |
| 19 | (GSK1278863):ti,ab |
| 20 | (AKB-6548):ti,ab |
| 21 | (JTZ-951):ti,ab |
| 22 | #1 OR #2 OR #3 OR #4 OR #5 OR #6 OR #7 OR #8 OR #9 OR #10 OR #11 OR #12 OR #13 OR #14 OR #15 OR #16 OR #17 OR #18 OR #19 OR #20 OR #21 |
| 23 | MeSH descriptor: [Renal Insufficiency, Chronic] explode all trees |
| 24 | MeSH descriptor: [Kidney Glomerulus] explode all trees |
| 25 | MeSH descriptor: [Kidney Diseases] explode all trees |
| 26 | “Chronic kidney disease”:ti,ab |
| 27 | CKD:ti,ab |
| 28 | “Chronic renal disease”:ti,ab |
| 29 | "end-stage kidney disease":ti,ab |
| 30 | "end-stage renal disease":ti,ab |
| 31 | ESRD:ti,ab |
| 32 | ESKD:ti,ab |
| 33 | ESKF:ti,ab |
| 34 | ESRF:ti,ab |
| 35 | "chronic renal failure":ti,ab |
| 36 | MeSH descriptor: [Renal Replacement Therapy] explode all trees |
| 37 | MeSH descriptor: [Sorption Detoxification] explode all trees |
| 38 | MeSH descriptor: [Renal Dialysis] explode all trees |
| 39 | MeSH descriptor: [Kidney Transplantation] explode all trees |
| 40 | Hemodialysis:ti,ab |
| 41 | "peritoneal dialysis":ti,ab |
| 42 | MeSH descriptor: [Ultrafiltration] 1 tree(s) exploded |
| 43 | “ultrafiltration”:ti,ab |
| 44 | Hemofiltration:ti,ab |
| 45 | Haemofiltration:ti,ab |
| 46 | Hemodiafiltration:ti,ab |
| 47 | Haemodiafiltration:ti,ab |
| 48 | Dialysis:ti,ab |
| 49 | Haemodialysis:ti,ab |
| 50 | Sorbtion:ti,ab |
| 51 | #23 OR #24 OR #25 OR #26 OR #27 OR #28 OR #25 OR #30 OR #31 OR #32 OR #33 OR #34 OR #35 OR #36 OR #37 OR #38 OR #39 OR #40 OR #41 OR #42 OR #43 OR #44 OR #45 OR #46 OR #47 OR #48 OR #49 OR #50 |
| 52 | #22 AND #51 |
| 53 | #64 AND #22 in trials only |

**Supplemental Figure 1. PRISMA diagram for identification of eligible publications for iron therapy review.**

**
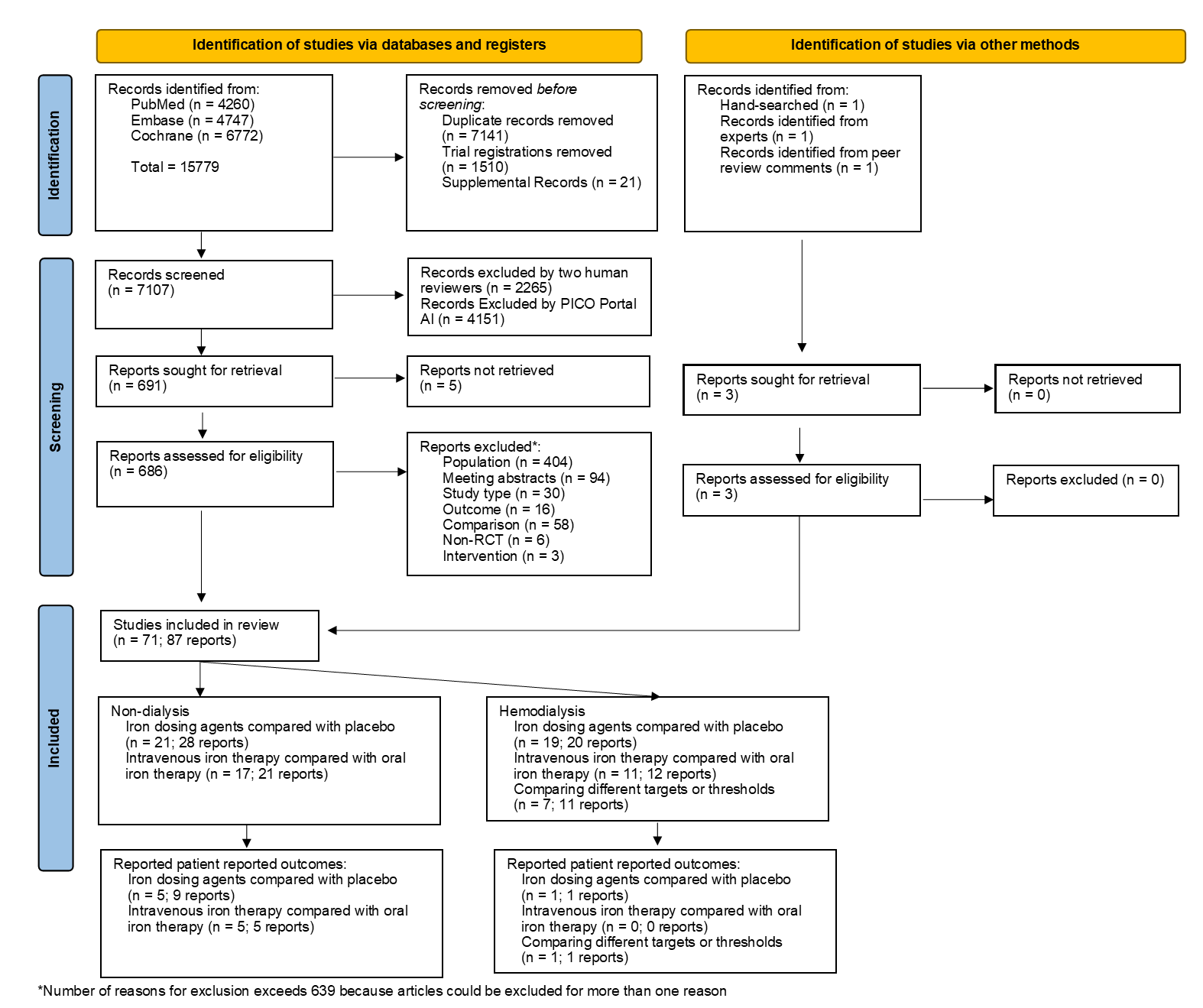
**

**Supplemental Figure 2. PRISMA diagram for identification of eligible publications for ESA review.** ESA: erythropoiesis stimulating agent.

**
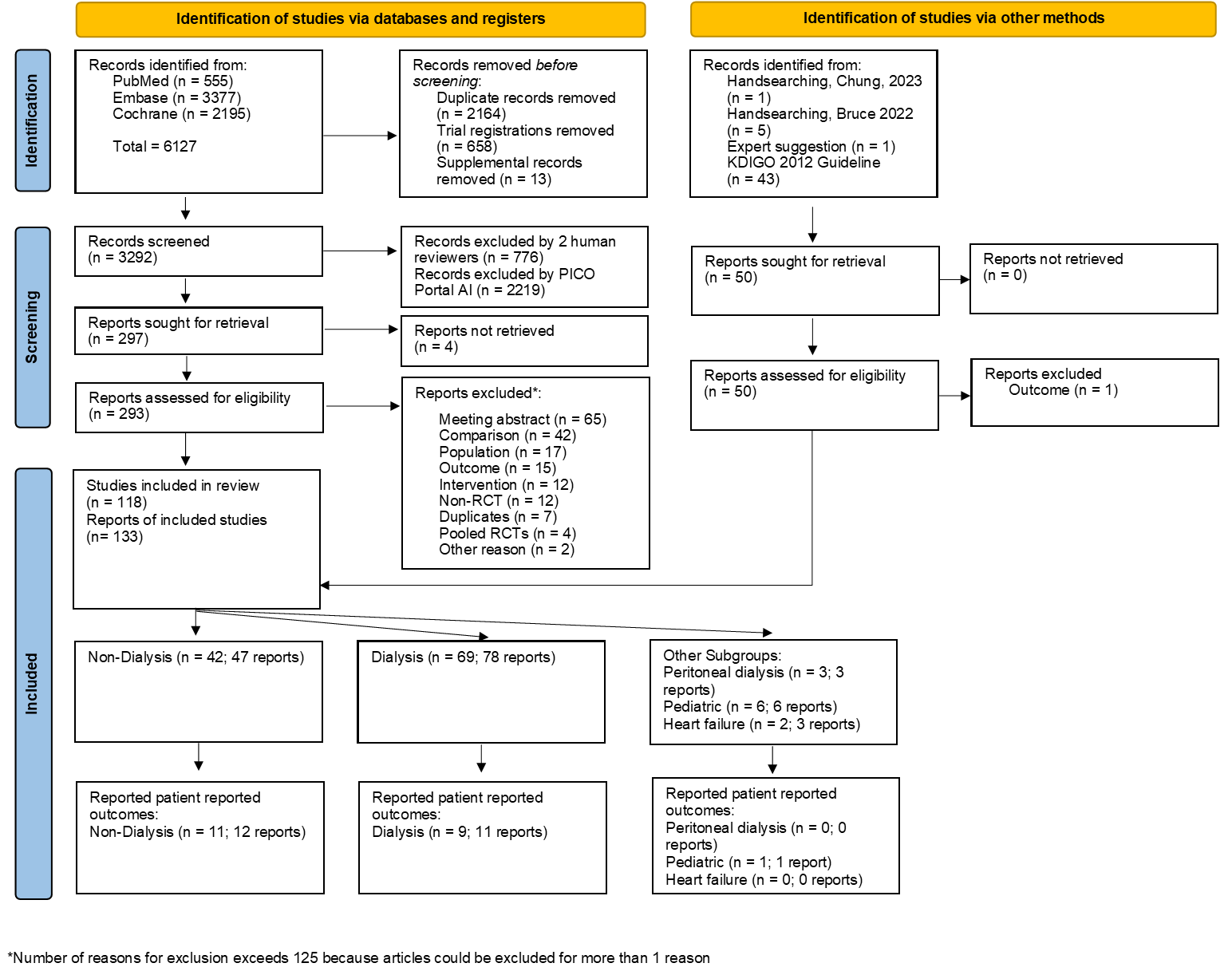
**

**Supplemental Figure 3. PRISMA diagram for identification of eligible publications for HIF-PHI review.** HIF-PHI: hypoxia-inducible factor prolyl hydroxylase inhibitors.

**
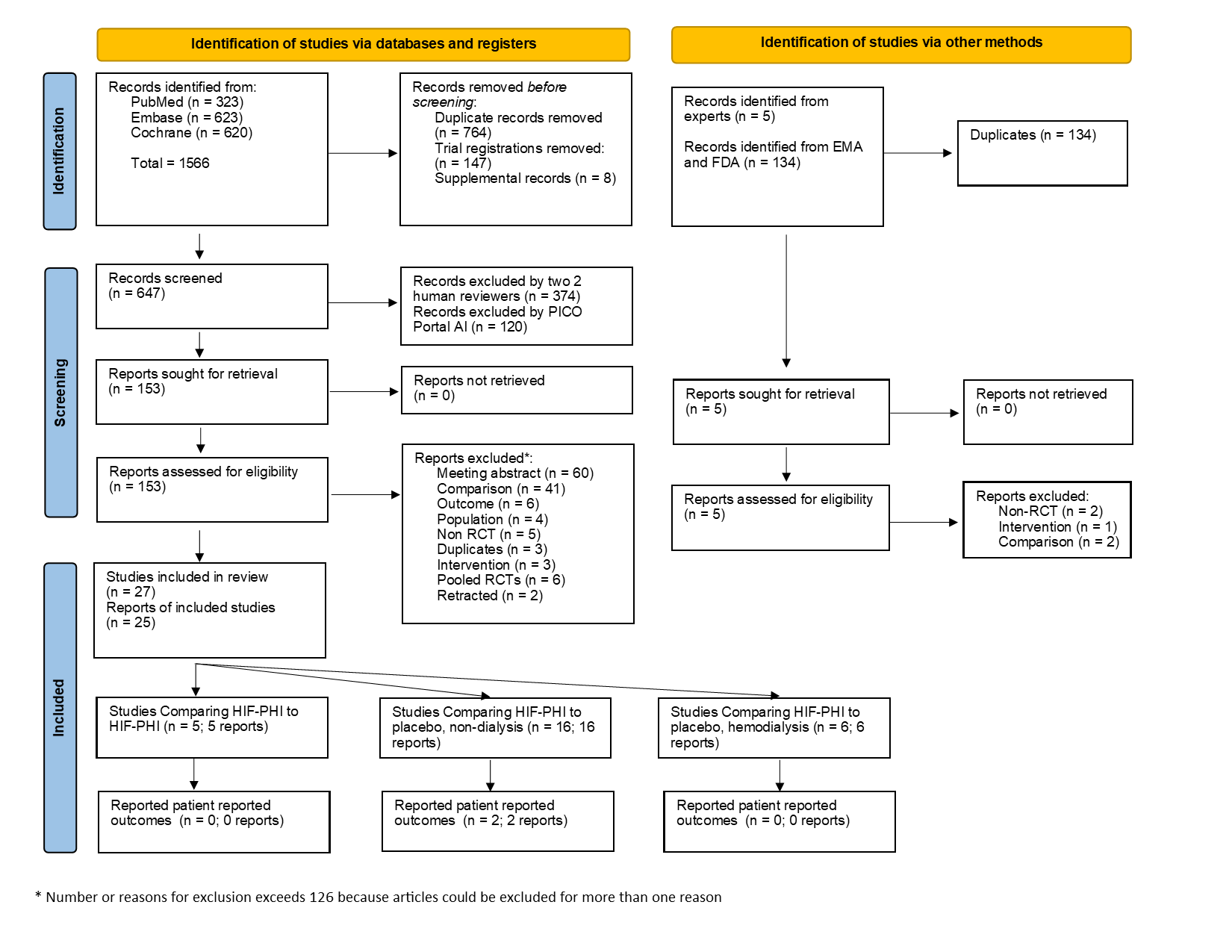
**

**Supplemental Figure 4. PRISMA diagram for identification of eligible publications for HIF-PHI versus ESA review.** ESA: erythropoiesis stimulating agent; HIF-PHI: hypoxia-inducible factor prolyl hydroxylase inhibitors.

**
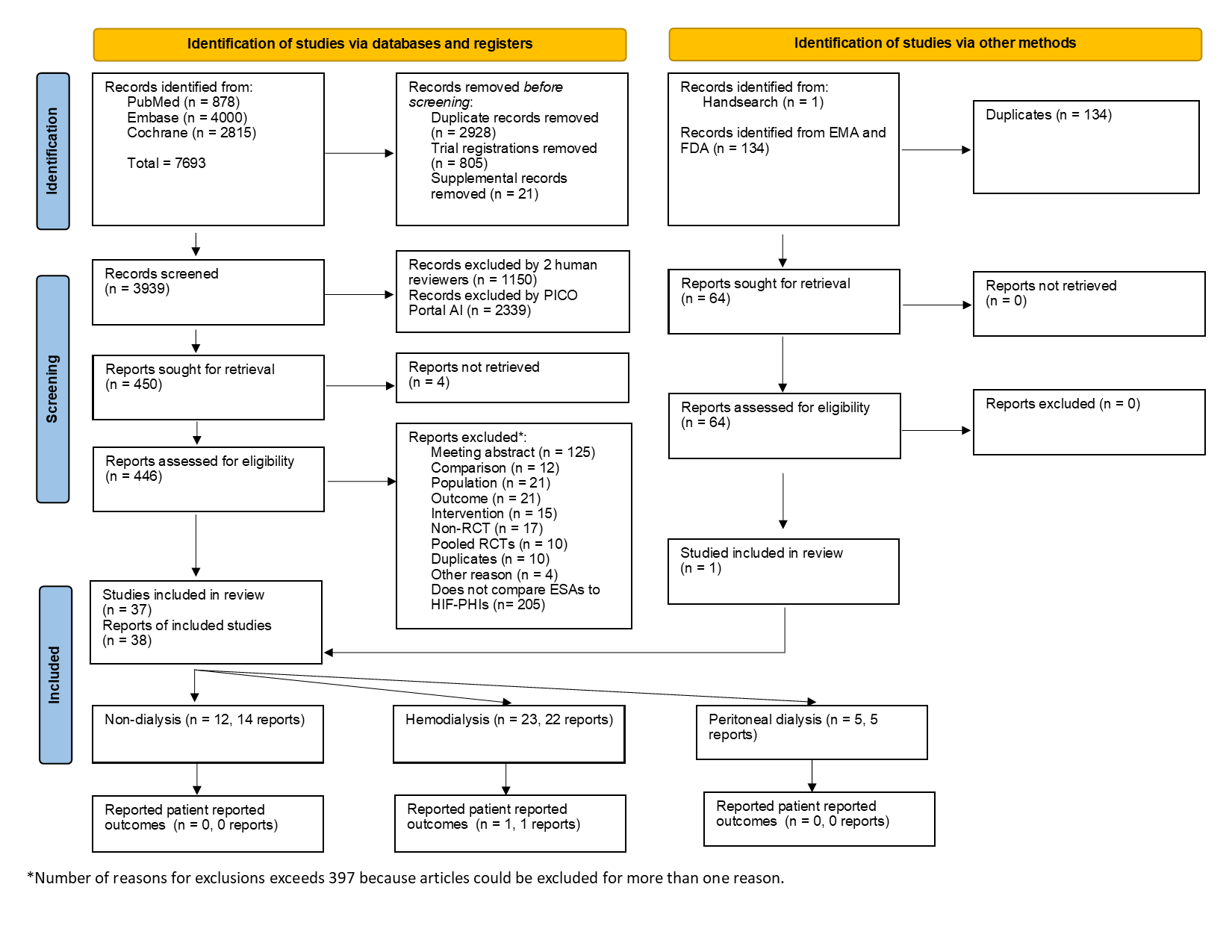
**

**Supplemental Table 10: Reporting of CONSORT-PRO specifications in publications reporting PROs data.** ESA: erythropoiesis stimulating agent; HIF-PHI: hypoxia-inducible factor prolyl hydroxylase inhibitors; PRO: patient-reported outcome.

| **Inter-vention** | **Publication** | **PRO measure (s) reported in study** | **Was PRO measure specified in abstract?** | **Was a rationale provided for use of the PRO measure?** | **Was a**  **hypothesis surround-ing PROs stated?** | **Were methods of PRO data collection specified?** | **Were statistical methods for handling missing PROs data provided?** | **Did the authors specify the number of PROs data at each time point?** | **Did authors discuss PRO-specific limitations and implications for generalizability?** | **# of elements reported (out of 7 elements of interest)** |
| --- | --- | --- | --- | --- | --- | --- | --- | --- | --- | --- |
| Iron | Van Wyck *et al.*, 2005 [1] | SF-36 | No | No | No | No | No | No | No | 0 |
|  | Agarwal *et al.*, 2006 [2] | KDQoL | Yes | Yes | No | No | No | No | No | 2 |
|  | Toblli *et al.*, 2007 [3] | MLHFQ, NYHA class | Yes | No | No | No | No | No | No | 1 |
|  | Anker *et al.*, 2009 [4, 5] | NYHA class, PGA | Yes | Yes | No | No | Yes | Yes | No | 4 |
|  | Comit-Colet *et al.*, 2013 [6] | KCCQ | Yes | Yes | No | No | Yes | No | No | 3 |
|  | Macdougall *et al.*, 2014 [7, 8] | SF-36 | No | No | No | No | No | No | No | 0 |
|  | Ponikowski *et al.*, 2015 [9] | EQ-5D | Yes | Yes | No | No | No | No | No | 2 |
|  | Agarwal *et al.*, 2015 [10] | KDQoL | No | No | No | No | No | Yes | No | 1 |
|  | Kalra *et al.*, 2016 [11] | LASA | No | No | No | No | No | Yes | No | 1 |
|  | Deng *et al.*, 2017 [12] | IRLS Rating Scale | Yes | Yes | No | No | No | Yes | No | 3 |
|  | Toblli *et al.,* 2017 [13] | NYHA class | Yes | No | No | No | No | No | No | 1 |
|  | Macdougall *et al.*, 2019 [14] | EQ-5D, KDQoL | No | No | No | No | Yes | Yes | No | 2 |
|  | Bhandari *et al.*, 2021 [15] | NYHA class, KDQoL-36, MLHFQ | No | Yes | Yes | No | No | Yes | No | 3 |
|  | Jankowska *et al.*, 2021 [16] | KCCQ-12 | Yes | Yes | No | Yes | Yes | Yes | Yes | 6 |
|  | Macdougall *et al.*, 2023 [17] | KCCQ-12 | No | Yes | No | No | No | No | No | 1 |
|  | Greenwood *et al.*, 2023 [18, 19] | Chalder fatigue, KDQOL-SF 1.3, WSAS | No | Yes | No | Yes | Yes | Yes | No | 4 |
| ESA | CanEPO study group [20] | SIP, TTO | Yes | Yes | No | Yes | Yes | Yes | Yes | 6 |
|  | Laupacis *et al.*, 1990 [21] | KCQ, SIP, TTO | n/a | Yes | No | No | No | No | No | 1 |
|  | Foley *et al.*, 2000 [22] | KDQ, SF-36, HUI | No | Yes | No | No | Yes | Yes | No | 3 |
|  | Furuland *et al.*, 2003 [23] | KDQ | Yes | Yes | No | No | No | Yes | No | 3 |
|  | Roger *et al.*, 2004 [24] | SF-36, RQoLP | No | No | No | No | No | No | No | 0 |
|  | Parfrey *et al.*, 2005 [25] | KDQoL/SF-36, FACIT | Yes | No | No | No | Yes | No | No | 2 |
|  | Rossert *et al.,* 2006 [26] | SF-36 | No | No | No | No | No | Yes | No | 1 |
|  | Drueke *et al.*, 2006 [27] | SF-36, NYHA class | No | No | No | No | No | No | No | 0 |
|  | Singh *et al.*, 2006 [28] | SF-36, KDQ, LASA | No | Yes | No | No | Yes | No | No | 2 |
|  | Ritz *et al.*, 2007 [29] | SF-36 | No | No | No | Yes | No | No | No | 1 |
|  | Foley *et al.*, 2009 [30] | KDQoL | Yes | Yes | Yes | No | No | No | No | 3 |
|  | Pfeffer *et al.*, 2009 [31] | FACT-fatigue | No | Yes | No | No | No | No | No | 1 |
|  | Choukroun *et al.*, 2010 [32] | KTQ-25, SF-36 | No | Yes | No | No | No | No | No | 1 |
|  | Akizawa *et al.*, 2011 [33] | SF-36, FACIT | No | No | No | No | No | No | No | 0 |
|  | Lewis *et al.*, 2011 [34] | FACT-fatigue, SF-36, EQ-5D | Yes | Yes | No | No | Yes | No | Yes | 4 |
|  | Roger *et al.*, 2014 [35] | SF-36, FACT-An | Yes | Yes | No | No | Yes | No | No | 3 |
|  | Oh *et al.*, 2014 [36] | SF-36 | No | No | No | No | No | No | No | 0 |
|  | Satirapoj *et al.*, 2014 [37] | SF-36 | No | No | No | No | No | No | No | 0 |
|  | Saglimbene *et al*., 2017 [38] | KDQOL-SF 1.3 | No | Yes | No | No | No | No | No | 1 |
|  | Satirapoj *et al.*, 2017 [39] | SF-36 | No | No | No | No | No | Yes | No | 1 |
|  | Warady *et al.*, 2018 [40] | PedsQL | Yes | Yes | No | No | No | Yes | Yes | 4 |
|  | Pile *et al.*, 2020 [41] | SF-36 | Yes | Yes | No | No | No | No | No | 2 |
| HIF-PHI | Fishbane *et al.*, 2021 [42] | SF-36 | No | No | No | No | No | No | No | 0 |
|  | Coyne *et al.*, 2021 [43] | SF-36 | No | No | Yes | No | No | No | No | 1 |
| HIF-PHI versus ESA | Csiky *et al.*, 2021 [44] | SF-36, EQ-5D-5I, PGIC, Fact-An | No | No | No | No | No | No | No | 0 |

EQ-5D: European Quality of Life 5-Dimension

FACIT-F: Functional Assessment of Chronic Illness Therapy: Fatigue

FACT-F: Functional Assessment of Cancer Therapy: Fatigue

Fact-An: Functional Assessment of Cancer Therapy: Anemia

HUI: Health Utility Index

IRLS: International Restless Legs Syndrome Study group Rating Scale for Restless Legs Syndrome

KCCQ: Kansas City Cardiomyopathy Questionnaire

KDQ: Kidney Disease Questionnaire

KDQoL: Kidney Disease Quality of Life

KTQ-25: Kidney Transplant Questionnaire-25

LASA: Linear Analogue Self-Assessment

MLHFQ: Minnesota Living With Heart Failure® Questionnaire

NYHA Class: New York Heart Association Classification
PedsQL: Pediatric Quality of Life

PGA: Patient Global Assessment

PGIC: Patient Global Impression of Change

RQLP: Renal Quality of Life Profile

SF-36: 36-Item Short-Form Survey

SIP: Sickness Impact Profile

TTO: Time Trade Off

WSAS: Work and Social Adjustment Scale

**Supplemental References**

1. Van Wyck, D.B., et al., *A randomized, controlled trial comparing IV iron sucrose to oral iron in anemic patients with nondialysis-dependent CKD.* Kidney Int, 2005. **68**(6): p. 2846-56.

2. Agarwal, R., et al., *A randomized controlled trial of oral versus intravenous iron in chronic kidney disease.* Am J Nephrol, 2006. **26**(5): p. 445-54.

3. Toblli, J.E., et al., *Intravenous iron reduces NT-pro-brain natriuretic peptide in anemic patients with chronic heart failure and renal insufficiency.* J Am Coll Cardiol, 2007. **50**(17): p. 1657-65.

4. Anker, S.D., et al., *Rationale and design of Ferinject assessment in patients with IRon deficiency and chronic Heart Failure (FAIR-HF) study: a randomized, placebo-controlled study of intravenous iron supplementation in patients with and without anaemia.* Eur J Heart Fail, 2009. **11**(11): p. 1084-91.

5. Anker, S.D., et al., *Ferric carboxymaltose in patients with heart failure and iron deficiency.* N Engl J Med, 2009. **361**(25): p. 2436-48.

6. Comin-Colet, J., et al., *The effect of intravenous ferric carboxymaltose on health-related quality of life in patients with chronic heart failure and iron deficiency: a subanalysis of the FAIR-HF study.* Eur Heart J, 2013. **34**(1): p. 30-8.

7. Macdougall, I.C., et al., *The FIND-CKD study--a randomized controlled trial of intravenous iron versus oral iron in non-dialysis chronic kidney disease patients: background and rationale.* Nephrol Dial Transplant, 2014. **29**(4): p. 843-50.

8. Macdougall, I.C., et al., *FIND-CKD: a randomized trial of intravenous ferric carboxymaltose versus oral iron in patients with chronic kidney disease and iron deficiency anaemia.* Nephrol Dial Transplant, 2014. **29**(11): p. 2075-84.

9. Ponikowski, P., et al., *The impact of intravenous ferric carboxymaltose on renal function: an analysis of the FAIR-HF study.* Eur J Heart Fail, 2015. **17**(3): p. 329-39.

10. Agarwal, R., J.W. Kusek, and M.K. Pappas, *A randomized trial of intravenous and oral iron in chronic kidney disease.* Kidney Int, 2015. **88**(4): p. 905-14.

11. Kalra, P.A., et al., *A randomized trial of iron isomaltoside 1000 versus oral iron in non-dialysis-dependent chronic kidney disease patients with anaemia.* Nephrol Dial Transplant, 2016. **31**(4): p. 646-55.

12. Deng, Y., J. Wu, and Q. Jia, *Efficacy of Intravenous Iron Sucrose in Hemodialysis Patients with Restless Legs Syndrome (RLS): A Randomized, Placebo-Controlled Study.* Med Sci Monit, 2017. **23**: p. 1254-1260.

13. Toblli, J. and F. Gennaro, *Long-Term Effect of Intravenous Iron on Overall Survival and Hospitalization in Patients with Heart Failure with Reduced Ejection Fraction, Iron Deficiency and Mild Renal Impairment: An Open-Label 5-Year Follow Up Observation.* Journal of Clinical and Diagnostic Research, 2017. **11**: p. OC18-OC24.

14. Macdougall, I.C., et al., *Intravenous Iron in Patients Undergoing Maintenance Hemodialysis.* New England Journal of Medicine, 2019. **380**(5): p. 447-458.

15. Bhandari, S., et al., *A multicentre prospective double blinded randomised controlled trial of intravenous iron (ferric Derisomaltose (FDI)) in Iron deficient but not anaemic patients with chronic kidney disease on functional status.* BMC Nephrol, 2021. **22**(1): p. 115.

16. Jankowska, E.A., et al., *The effect of intravenous ferric carboxymaltose on health-related quality of life in iron-deficient patients with acute heart failure: the results of the AFFIRM-AHF study.* Eur Heart J, 2021. **42**(31): p. 3011-3020.

17. Macdougall, I.C., et al., *Ferric Carboxymaltose in Iron-Deficient Patients with Hospitalized Heart Failure and Reduced Kidney Function.* Clin J Am Soc Nephrol, 2023. **18**(9): p. 1124-1134.

18. Greenwood, S.A., et al., *The effect of intravenous iron supplementation on exercise capacity in iron-deficient but not anaemic patients with chronic kidney disease: study design and baseline data for a multicentre prospective double-blind randomised controlled trial.* BMC Nephrol, 2022. **23**(1): p. 268.

19. Greenwood, S.A., et al., *A Randomized Trial of Intravenous Iron Supplementation and Exercise on Exercise Capacity in Iron-Deficient Nonanemic Patients With CKD.* Kidney Int Rep, 2023. **8**(8): p. 1496-1505.

20. *Association between recombinant human erythropoietin and quality of life and exercise capacity of patients receiving haemodialysis. Canadian Erythropoietin Study Group.* BMJ, 1990. **300**(6724): p. 573-8.

21. Laupacis, A., *Changes in quality of life and functional capacity in hemodialysis patients treated with recombinant human erythropoietin. The Canadian Erythropoietin Study Group.* Semin Nephrol, 1990. **10**(2 Suppl 1): p. 11-9.

22. Foley, R.N., et al., *Effect of hemoglobin levels in hemodialysis patients with asymptomatic cardiomyopathy.* Kidney Int, 2000. **58**(3): p. 1325-35.

23. Furuland, H., et al., *A randomized controlled trial of haemoglobin normalization with epoetin alfa in pre-dialysis and dialysis patients.* Nephrol Dial Transplant, 2003. **18**(2): p. 353-61.

24. Roger, S.D., et al., *Effects of early and late intervention with epoetin alpha on left ventricular mass among patients with chronic kidney disease (stage 3 or 4): results of a randomized clinical trial.* J Am Soc Nephrol, 2004. **15**(1): p. 148-56.

25. Parfrey, P.S., et al., *Double-blind comparison of full and partial anemia correction in incident hemodialysis patients without symptomatic heart disease.* J Am Soc Nephrol, 2005. **16**(7): p. 2180-9.

26. Rossert, J., et al., *Effect of early correction of anemia on the progression of CKD.* Am J Kidney Dis, 2006. **47**(5): p. 738-50.

27. Drueke, T.B., et al., *Normalization of hemoglobin level in patients with chronic kidney disease and anemia.* N Engl J Med, 2006. **355**(20): p. 2071-84.

28. Singh, A.K., et al., *Correction of anemia with epoetin alfa in chronic kidney disease.* N Engl J Med, 2006. **355**(20): p. 2085-98.

29. Ritz, E., et al., *Target level for hemoglobin correction in patients with diabetes and CKD: primary results of the Anemia Correction in Diabetes (ACORD) Study.* Am J Kidney Dis, 2007. **49**(2): p. 194-207.

30. Foley, R.N., B.M. Curtis, and P.S. Parfrey, *Erythropoietin therapy, hemoglobin targets, and quality of life in healthy hemodialysis patients: a randomized trial.* Clin J Am Soc Nephrol, 2009. **4**(4): p. 726-33.

31. Pfeffer, M.A., et al., *A trial of darbepoetin alfa in type 2 diabetes and chronic kidney disease.* N Engl J Med, 2009. **361**(21): p. 2019-32.

32. Choukroun, G., et al., *Correction of postkidney transplant anemia reduces progression of allograft nephropathy.* J Am Soc Nephrol, 2012. **23**(2): p. 360-8.

33. Akizawa, T., et al., *Positive outcomes of high hemoglobin target in patients with chronic kidney disease not on dialysis: a randomized controlled study.* Ther Apher Dial, 2011. **15**(5): p. 431-40.

34. Lewis, E.F., et al., *Darbepoetin alfa impact on health status in diabetes patients with kidney disease: a randomized trial.* Clin J Am Soc Nephrol, 2011. **6**(4): p. 845-55.

35. Roger, S.D., et al., *A randomised single-blind study to improve health-related quality of life by treating anaemia of chronic kidney disease with Aranesp(R) (darbepoetin alfa) in older people: STIMULATE.* Int Urol Nephrol, 2014. **46**(2): p. 469-75.

36. Oh, J., et al., *Correction of anemia with continuous erythropoietin receptor activator in Korean patients on long-term hemodialysis.* J Korean Med Sci, 2014. **29**(1): p. 76-83.

37. Satirapoj, B., O. Supasyndh, and P. Choovichian, *A comparative study of efficacy and safety of the lyophilized powder alpha-erythropoietin and the liquid form alpha-erythropoietin for hemoglobin maintenance in patients with hemodialysis treatment.* J Med Assoc Thai, 2014. **97**(9): p. 899-906.

38. Saglimbene, V., et al., *Low versus high dose erythropoiesis-stimulating agents in hemodialysis patients with anemia: A randomized clinical trial.* PLoS One, 2017. **12**(3): p. e0172735.

39. Satirapoj, B., R. Dispan, and O. Supasyndh, *Efficacy and safety of subcutaneous administration of lyophilized powder of alfa-erythropoietin to maintain hemoglobin concentrations among hemodialysis patients.* Int J Nephrol Renovasc Dis, 2017. **10**: p. 275-283.

40. Warady, B.A., et al., *De novo weekly and biweekly darbepoetin alfa dosing in pediatric patients with chronic kidney disease.* Pediatr Nephrol, 2018. **33**(1): p. 125-137.

41. Pile, T., et al., *Treating Posttransplant Anemia With Erythropoietin Improves Quality of Life but Does Not Affect Progression of Chronic Kidney Disease.* Exp Clin Transplant, 2020. **18**(1): p. 27-33.

42. Fishbane, S., et al., *Roxadustat for Treating Anemia in Patients with CKD Not on Dialysis: Results from a Randomized Phase 3 Study.* J Am Soc Nephrol, 2021. **32**(3): p. 737-755.

43. Coyne, D.W., et al., *Roxadustat for CKD-related Anemia in Non-dialysis Patients.* Kidney Int Rep, 2021. **6**(3): p. 624-635.

44. Csiky, B., et al., *Roxadustat for the Maintenance Treatment of Anemia in Patients with End-Stage Kidney Disease on Stable Dialysis: A European Phase 3, Randomized, Open-Label, Active-Controlled Study (PYRENEES).* Adv Ther, 2021. **38**(10): p. 5361-5380.
